# Biosignature Preservation in Stromatolites From the Carri Laufquen Lakes System, Patagonia: Implications for Martian Palaeolacustrine Environments

**DOI:** 10.1111/gbi.70063

**Published:** 2026-09-09

**Authors:** Victor Amir Cardoso Dorneles, Maëva Millan, Fernando J. Gómez, Primož Šket, Jens Najorka, Cindy Broderick, Wren Montgomery, Barbara Cavalazzi, Keyron Hickman‐Lewis

**Affiliations:** ^1^ Department of Biological, Geological and Environmental Sciences University of Bologna Bologna Italy; ^2^ Sedimentary Geology Laboratory Federal University of Rio de Janeiro Rio de Janeiro Brazil; ^3^ Laboratoire Atmosphère, Observations Spatiales (LATMOS), LATMOS/IPSL, UVSQ Université Paris‐Saclay, Sorbonne Université, CNRS Guyancourt France; ^4^ CICTERRA‐CONICET Universidad Nacional de Córdoba Córdoba Argentina; ^5^ Slovenian NMR Centre National Institute of Chemistry Ljubljana Slovenia; ^6^ The Natural History Museum London UK; ^7^ Department of Geology University of Johannesburg Johannesburg South Africa; ^8^ School of Natural Sciences Birkbeck, University of London London UK

**Keywords:** Al‐silicate, biosignatures, Mars sample return, Mg‐carbonate, microbialites, taphonomy

## Abstract

The Carri Laufquen Lakes System is a highly alkaline, basalt‐hosted lacustrine environment in northern Patagonia, Argentina. Diverse morphologies of Late Pleistocene sub‐fossil stromatolites occur along the lakeshores. Using detailed optical and electron microscopy, spectroscopy and organic geochemical analyses, this study shows that stromatolite growth and early diagenetic processes were strongly influenced by microbially controlled organomineralisation. Mineralisation of extracellular polymeric substances and cyanobacterial sheaths promoted early lithification and exceptional preservation of stromatolite fabrics and biosignatures. Organic matter is closely associated with Al–bearing silicate minerals at the micro–nanoscale, enhancing fossilisation potential and preserving biogeochemical signatures. The resulting mineral assemblage in the Carri Laufquen stromatolites, dominated by Mg‐carbonate with minor Al‐silicates and carbonaceous material, resembles the geochemistry of ancient lacustrine deposits around the margins of Jezero crater, highlighting the propensity of such deposits for biosignature preservation, and providing a framework for detailed characterisation of such materials following Mars Sample Return.

## 1 Introduction

Stromatolites are layered organosedimentary structures formed by interactions between benthic microbial communities and chemical and/or trapped sediments (Burne and Moore 1987; Bosak et al. 2013; Hickman‐Lewis et al. 2019). As archetypal microbe–sediment systems, they illustrate how biological activity and physicochemical processes jointly influence complex sedimentary architectures (Suosaari et al. 2016; Barbieri 2026). As macroscale biosignatures, stromatolites also preserve a three‐dimensional record of ecosystem–environment interplays over short and long geological timescales (e.g., Kah et al. 2009; Bosak et al. 2013; Hickman‐Lewis et al. 2023).

Stromatolites are among the oldest evidence for life on Earth, with a fossil record extending back to at least the Palaeoarchaean. Ancient stromatolites, such as those in the East Pilbara, Western Australia, provide critical insights into early ecosystems and their environments (Walter et al. 1980; Hickman‐Lewis et al. 2023). Once widespread across Earth's surface, living stromatolites are now largely confined to extreme environments, including highly alkaline lakes (e.g., Çağatay et al. 2024; Dorneles et al. 2024, 2026). In the high‐altitude subtropical arid, semi‐arid and tropical Andes of southern South America, lacustrine stromatolites have developed under extreme and highly variable environmental conditions (Pacton et al. 2015). Stromatolites in Patagonia and the Andean Puna plateau often occur along (palaeo)lake shorelines, serving as archives of hydrological and climatic changes (e.g., Bradbury et al. 2001; Tatur et al. 2002). Although fossil stromatolites are widespread throughout Patagonia, active stromatolite formation in modern Andean lakes has also been observed, where microbial communities persist under extreme environmental conditions characterised by high UV radiation, elevated alkalinity, high arsenic and salt concentrations, and low atmospheric O_2_ (e.g., Farías et al. 2013; Gomez et al. 2014; Buongiorno et al. 2019).

This work presents a detailed investigation of sub‐fossil stromatolites from the Carri Laufquen Lakes System (Maquinchao Basin, Patagonia, Argentina), a highly alkaline, basalt‐hosted lacustrine environment. A multi‐analytical approach was used to characterise stromatolitic microfacies, examining microbial influences on micromorphology and microstructure and evaluating their potential for biosignature preservation. By improving understanding of organomineralisation and microbial biosignature preservation in alkaline lacustrine environments, these findings improve the use of terrestrial analogues for evaluating potential Martian palaeohabitats, provide guidance for in situ biosignature searches on Mars and allow the development of fossilisation models relevant to the study of materials brought to Earth by Mars Sample Return (MSR) missions.

## 2 Geological Setting

**FIGURE 1:**
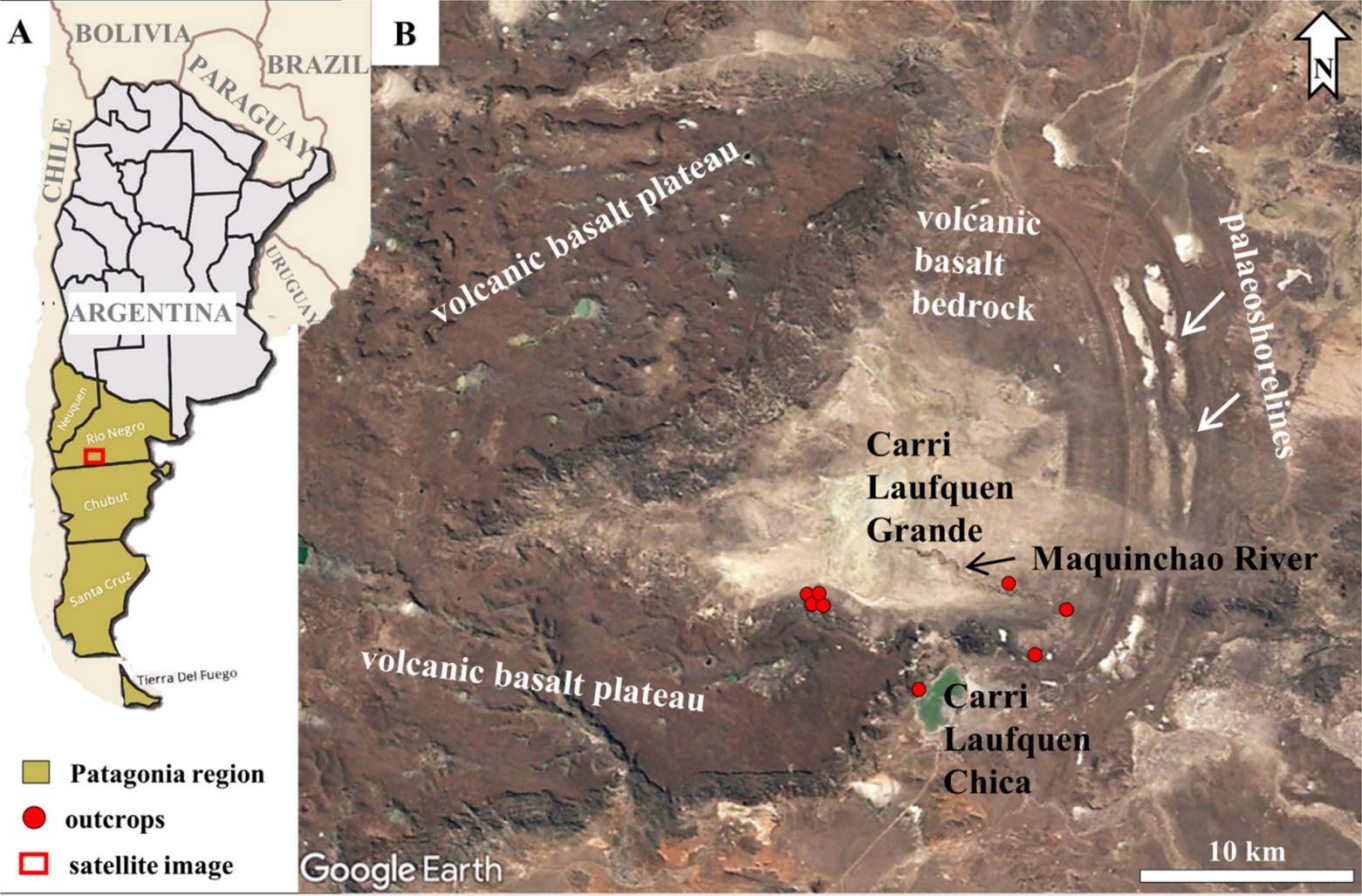
Geographical and geological context of the Carri Laufquen Lakes System. (A) Map showing the location of the Carri Laufquen Lakes System (Rio Negro, Patagonia, Argentina). (B) Satellite image showing the association between lacustrine deposits with volcanic basalt plateaux. During fieldwork in March 2023, Carri Laufquen Grande was completely dry and Carri Laufquen Chica was grass‐covered. Satellite image from Landsat Copernicus (2020), via Google Earth.

The Maquinchao Basin is an endorheic basin in northern Patagonia (Río Negro, Argentina; 41°80 47″ S, 69°270 37″ W), comprising two main lakes: Carri Laufquen Grande (CLG) and Carri Laufquen Chica (CLC) (Pacton et al. 2015). The lakes are located on dark, olivine‐rich plateau basalts of the *meseta* Cari Laufquen, dated to Late Oligocene–Early Miocene (Aguilera et al. 2018), which forms part of the volcanic plateau areas surrounding the Maquinchao Basin. This tectonic depression is bounded to the west and east by Mesozoic to Tertiary basaltic sequences exceeding 1000 m in elevation (figure 1; Pacton et al. 2015; Aguilera et al. 2018; Alvarez et al. 2022). The basin developed over a succession of Mesozoic volcanic rocks, clays, calcareous sandstones, conglomerates, and Miocene volcanic deposits (Whatley and Cusminsky 1999). The uppermost Pleistocene strata are dominated by fluvial and lacustrine deposits, including stromatolitic carbonates.

The region has a semi‐arid climate with a mean annual temperature of ~10°C, ranging between 16.1°C and 1.7°C during Austral summer and winter, respectively, and with a mean annual precipitation of ~200 mm (Pacton et al. 2015). CLG (781 m a.s.l.) spans 18.2 km^2^ with water depths of 8–10 m and a pH of 8.8; CLC (821 m a.s.l.) covers 5.5 km^2^ with similar water depths and a pH of 8.7 (Eymard et al. 2019). During our fieldwork—conducted in March 2023—both lakes were dry and unconnected by the Maquinchao River, in contrast to conditions reported in Pacton et al. (2015) and Eymard et al. (2021). The entire lakebed of CLC was covered by grasses (Figure 1), suggesting the presence of localised near‐surface sediment‐hosted waters. Although lake‐level fluctuations and episodic desiccation are recurrent features of the Maquinchao Basin, recent years have been characterised by particularly severe drying, culminating in the complete desiccation of CLG (Alvarez et al. 2022).

Satellite observations indicate a recent decline in water levels in both CLG and CLC; however, palaeoshorelines along the eastern margin of CLG, dated to 19–8 ka, record the former presence of a large, high‐altitude palaeolake formed by the post‐Pleistocene coalescence of the two basins, covering more than 1500 km^2^ (Galloway et al. 1988; Alvarez et al. 2022). Multiple paleoclimate studies suggest elevated lake levels during this interval. Fossil stromatolites have been correlated with distinct beach‐ridge deposits and three palaeolake stillstands between 820 and 830 m elevation, indicating that large‐scale microbialite growth occurred during discrete phases of lake‐level stability between ~19 and 22 ka BP (Eymard et al. 2019; Alvarez et al. 2022). Fine‐grained sediments associated with these paleoshorelines, rich in diatoms and ostracods, are also consistent with deposition in an alkaline lacustrine environment (Whatley and Cusminsky 1999).

Previous studies have documented carbonate accumulations in the Carri Laufquen Lakes System, often described as tufa with globular, flower‐like morphologies (Cartwright et al. 2011). These laminated carbonate structures typically form around basaltic cores (e.g., pebbles, cobbles, and boulders) and exhibit evidence of directional growth. Steep basaltic escarpments along the southern lake margin supplied boulder‐sized substrates, whereas lower‐relief areas are characterised by pebble‐sized basaltic clasts (Eymard et al. 2019). Similar carbonate build‐ups have also been observed in actively forming microbialites along the nearby Maquinchao River (Pacton et al. 2015).

## 3 Results

### 3.1 Field and Outcrop Observations

**FIGURE 2:**
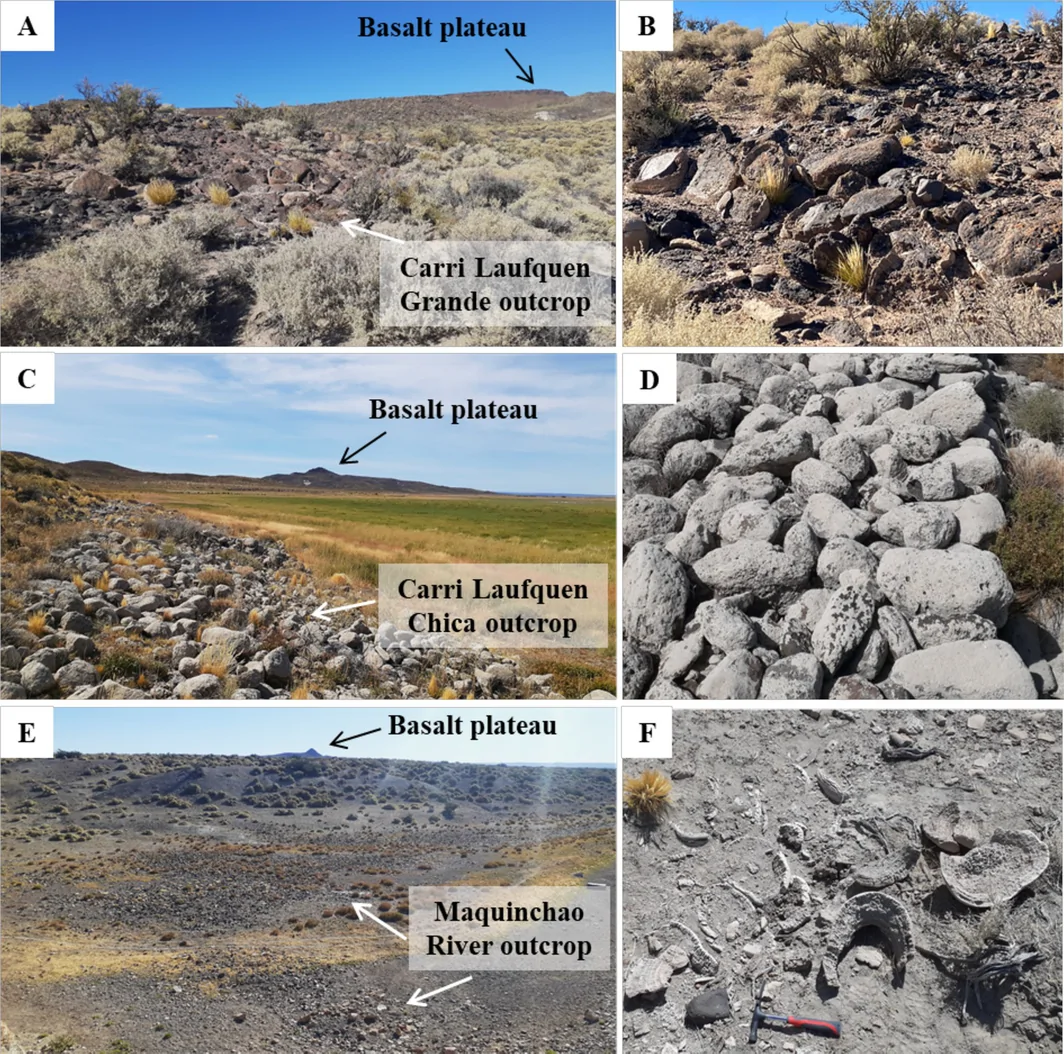
Overview of studied outcrops and sampling localities. (A) Carri Laufquen Grande (CLG) outcrop at the base of a basalt plateau. (B) Close‐up of the dm‐scale pebbles with stromatolitic crusts. (C) Carri Laufquen Chica (CLC) outcrop on the western lake shores. (D) Close‐up of cm‐scale pebbles with mm‐thick carbonate coatings. (E) Maquinchao River outcrop. (F) Fragmented stromatolite heads with layered structures similar to those at CLG.

Stromatolitic outcrops in CLG occur along the south–southwest margin of the former lake, at the base of the surrounding basalt plateaux, between 822 and 830 m in elevation (Figure 2A,B), coinciding with palaeoshoreline deposits previously recognised in the basin (Eymard et al. 2019; Alvarez et al. 2022). In CLC, stromatolitic deposits are limited to thin carbonate coatings covering pebbles along the western shore (Figure 2C,D). Additional stromatolitic outcrops occur along both banks of the presently dry Maquinchao River, within the same elevation range (Figure 2E,F).

**FIGURE 3:**
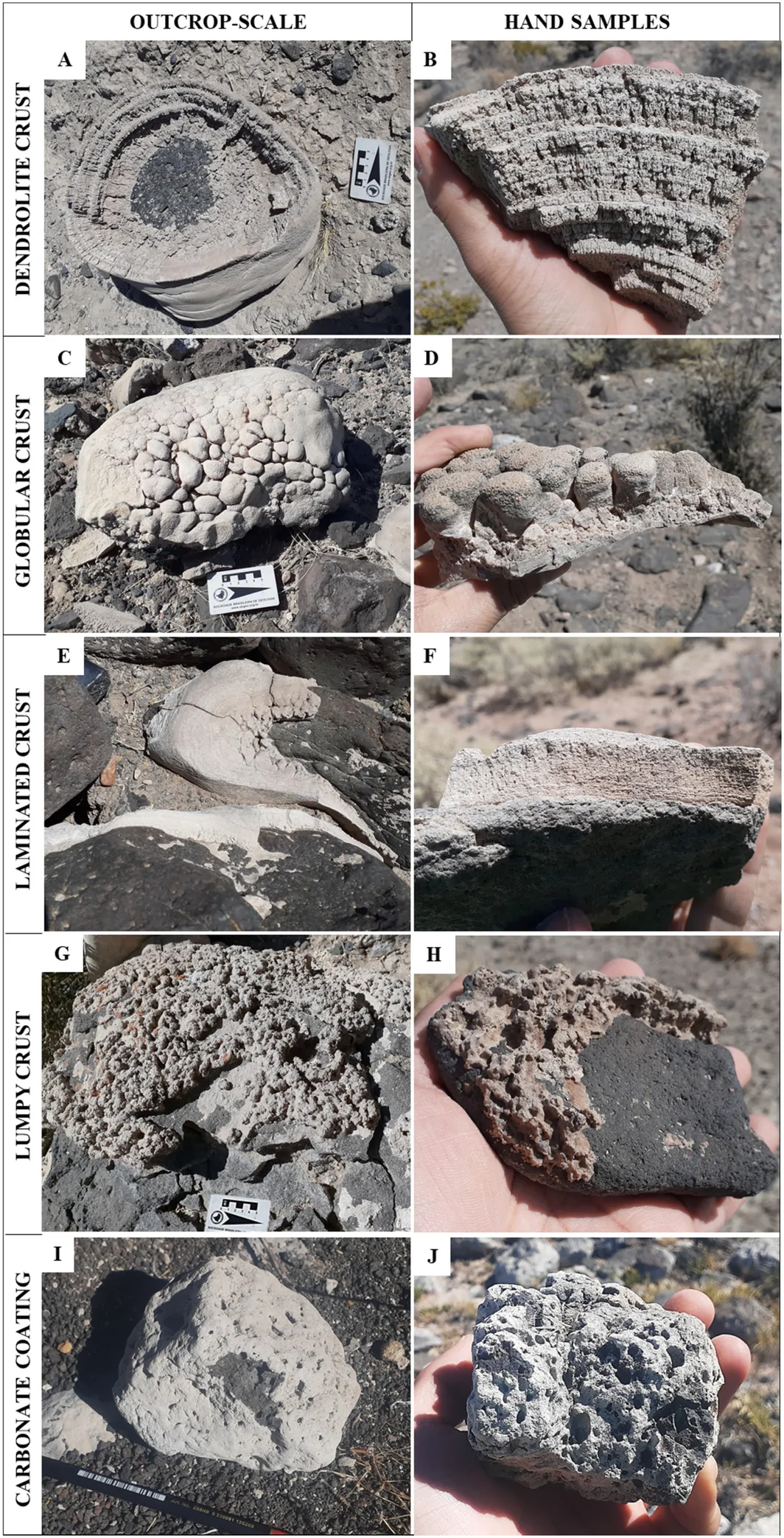
Field photographs showing distinct stromatolite morphologies in outcrop and hand sample. (A, B) Dendrolite crust, composed of layers of shrub‐like microcolumnar structures. (C, D) Globular crust, composed of cerebroid columns. (E, F) Laminated crust with very flat and smooth layered structure. (G, H) Lumpy crust, characterised by irregular carbonate precipitations. (I, J) Carbonate coatings comprising very thin and friable sub‐mm carbonate layers covering basalt pebbles.

Typical outcrops consist of dispersed, rounded basalt pebbles to boulders (up to 1 m in diameter) derived from the surrounding Miocene basaltic plateaux and coated by stromatolitic carbonate crusts, millimetres–centimetres in thickness, that grow outward from the basaltic substrate (Figure 2). In CLG, these stromatolite‐bearing deposits occur within a succession reaching thicknesses of up to ~4 m. Stromatolites are often well preserved in situ as complete structures surrounding individual basalt pebbles (e.g., Figures 2B and 3A) but may also occur as partially eroded and fragmented portions, reflecting the detachment of stromatolitic crusts from basalt blocks (Figure 2F).

Five distinct stromatolite macromorphologies were identified across the studied areas of the Maquinchao Basin, each defining a stromatolitic facies (Figure 3). These morphologies commonly co‐occur within individual stromatolite build‐ups and throughout the same outcrop, with gradual transitions between them suggesting successive stages of stromatolitic crust development.

#### 3.1.1 Dendrolite Crust

The most common and thickest facies (5–50 cm), characterised by well‐defined dendritic laminae (< 1 cm thick) forming shrub‐ or tree‐like architectures (*sensu* Jones and Renaut 2008; Figure 3A,B). The upper growth surface is typically flat and smooth.

#### 3.1.2 Globular Crust

Crusts composed of unbranched, globular and cerebroid columns (*sensu* Pacton et al. 2015), typically 1–3 cm thick and up to 5 cm in diameter. Columns are loosely packed and separated by millimetre‐scale intercolumnar voids, producing a macroporous texture (Figure 3C,D).

#### 3.1.3 Laminated Crust

The rarest facies, consisting of smooth, millimetric, flat laminae forming crusts between a few mm and ~6 cm thick, and with a flat external surface (Figure 3E,F).

#### 3.1.4 Lumpy Crust

Thin (< 1 cm), irregular carbonate layers that partly cover basaltic pebbles and sometimes transition into poorly developed columnar morphologies (Figure 3G,H).

#### 3.1.5 Carbonate Coating

Thin (< 1 mm), friable carbonate layers enveloping cm‐ to dm‐sized pebbles, which are particularly abundant at CLC. Vesicular basalts also often feature carbonate infillings of surface vesicles (Figure 3I,J).

### 3.2 Petrographical, Mineralogical and Chemical Characterisation of Stromatolites

Despite the delineation of five macromorphologies in outcrop, petrographic observations identified only three microfacies. These distinct microfacies commonly co‐occur within individual samples and typically exhibit microstratigraphic variation.

#### 3.2.1 Laminated Microsparite Microfacies

**FIGURE 4:**
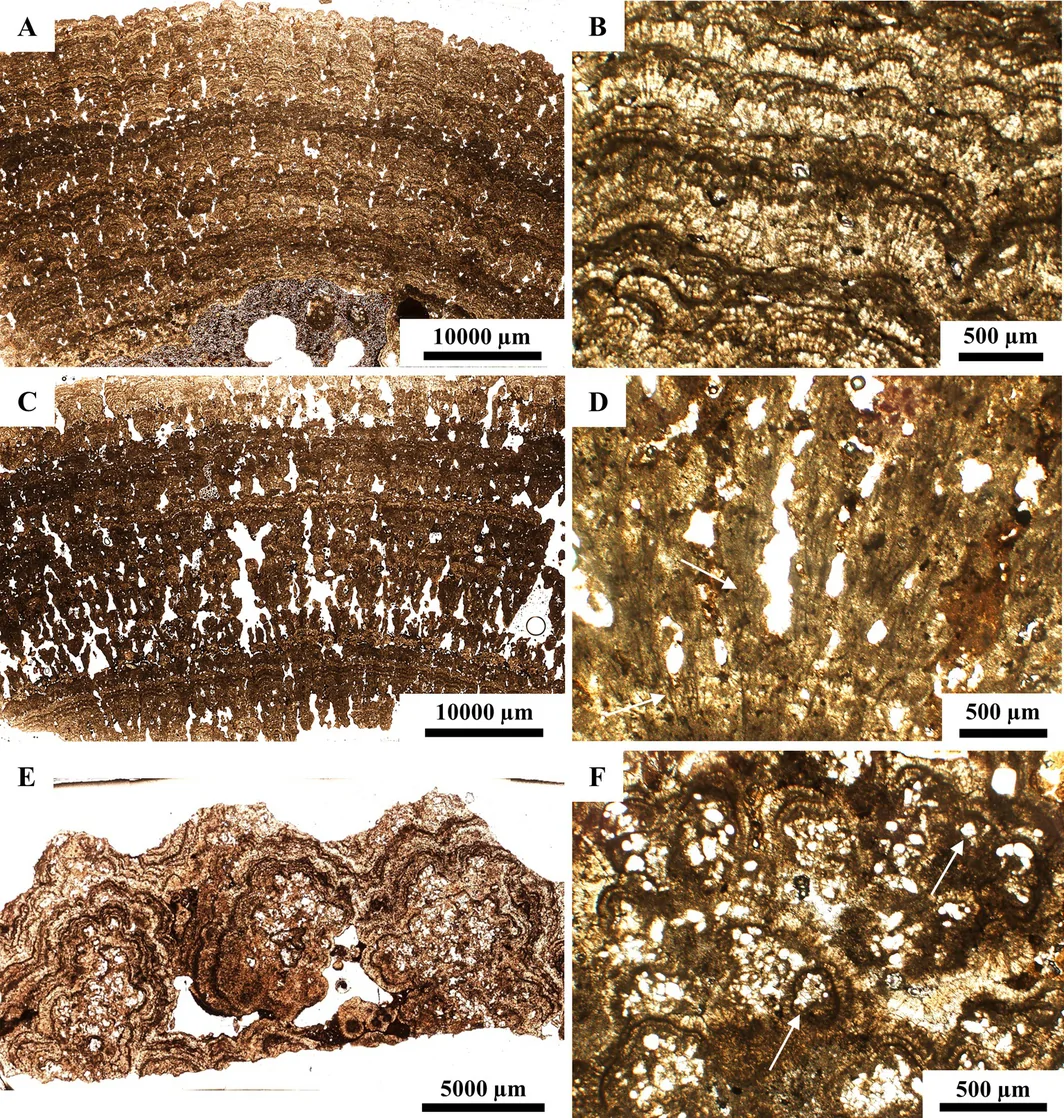
Photomicrographs showing the three distinct Carri Laufquen stromatolite microfacies. (A) Overview image of the laminated microsparite microfacies. (B) Detail showing well‐preserved lamination in laminated microsparite microfacies composed of interbedded bright microsparite laminae and dark thin micrite laminae. (C) Overview image of the micritic columnar microfacies. (D) Detail of microcolumns in the micritic columnar microfacies showing the presence of filamentous structures (examples arrowed) associated with micritic carbonate. (E) Overview image of the botryoidal microfacies. (F) Detail of the irregular structures formed by botryoidal coalescence, showing nuclei of calcite grains (examples arrowed).

Dominated by low‐porosity radial–fibrous low‐Mg calcite forming microsparitic laminae, interbedded with thin micritic laminae. Laminae are upward‐convex, locally coalescing into microcolumns (Figure 4A,B). Filamentous microstructures are rare.

#### 3.2.2 Micritic Columnar Microfacies

Composed of unlaminated micritic columns containing vertically oriented, well‐preserved filamentous structures (Figure 4C,D). The porosity is higher than in the laminated microsparite facies.

#### 3.2.3 Botryoidal Microfacies

Characterised by concentric microsparite and micrite laminae surrounding calcitic grains, forming botryoid‐like aggregates (Figure 4E,F). Filamentous microstructures are preferentially preserved in micrite layers.

Clay minerals occur in all samples; however, their abundance and microtextural expression vary between microfacies. Rare diatom frustules are also present in most samples. Detrital grains, including quartz, plagioclase, pyroxene, and clay, occur in all microfacies and are often trapped within microstructural voids. Evidence for early diagenesis is most pronounced at the basalt–carbonate interface, where peloid‐like texture develops and micritisation locally affects botryoidal nuclei.

**FIGURE 5:**
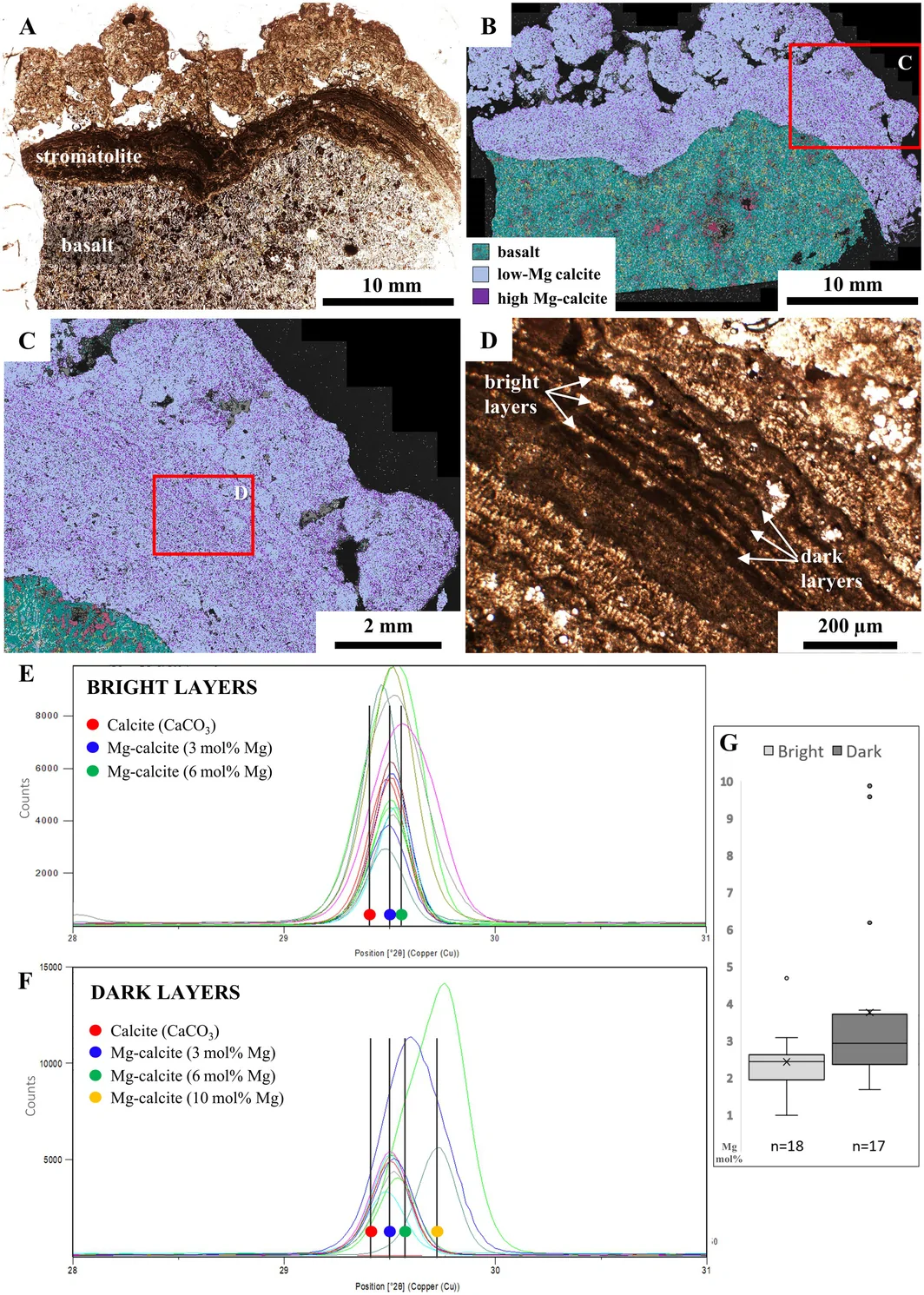
Mineralogical composition of the Carri Laufquen stromatolites. (A) Optical photomicrograph showing sequential stromatolitic microfacies: The laminated microsparitic microfacies in contact with the basalt pebble, showing well‐defined lamination, is overlain by the botryoidal microfacies. (B) TESCAN TIMA phase map showing mineralogical variation within the sample. (C) Close‐up image showing an intercalation of low‐Mg calcite and high‐Mg calcite phases in the stromatolite. (D) Optical photomicrograph of the region of interest highlighted in C, showing that compositional variation in calcite phases with higher or lower Mg content is expressed in the colour variation of the stromatolitic laminae. (E–G) μXRD patterns (E, F) and boxplot diagram (G) showing variation in Mg between the light and dark laminae.

Micro X‐ray diffraction (μXRD) analyses and TESCAN Integrated Mineral Analyzer (TIMA) phase mapping identified calcite as the dominant carbonate mineral in the Carri Laufquen stromatolites. Based on Mg contents derived from the position of the calcite d104 reflection in μXRD patterns, two compositional phases were distinguished: low‐Mg calcite and high‐Mg calcite. This compositional variation is expressed optically as alternations between bright and dark laminae: bright laminae are composed predominantly of low‐Mg calcite (1.0–4.7 mol% Mg, avg. 2.4 mol%), whereas dark laminae are enriched in high‐Mg calcite (1.7–10.0 mol% Mg, avg. 3.8 mol%) (Figure 5A–G). Although individual μXRD measurements show some overlap in Mg contents (Figure 5E,F), dark laminae consistently exhibit higher average Mg concentrations than bright laminae (Figure 5G), indicating distinct compositional variations rather than discrete compositional boundaries.

**FIGURE 6:**
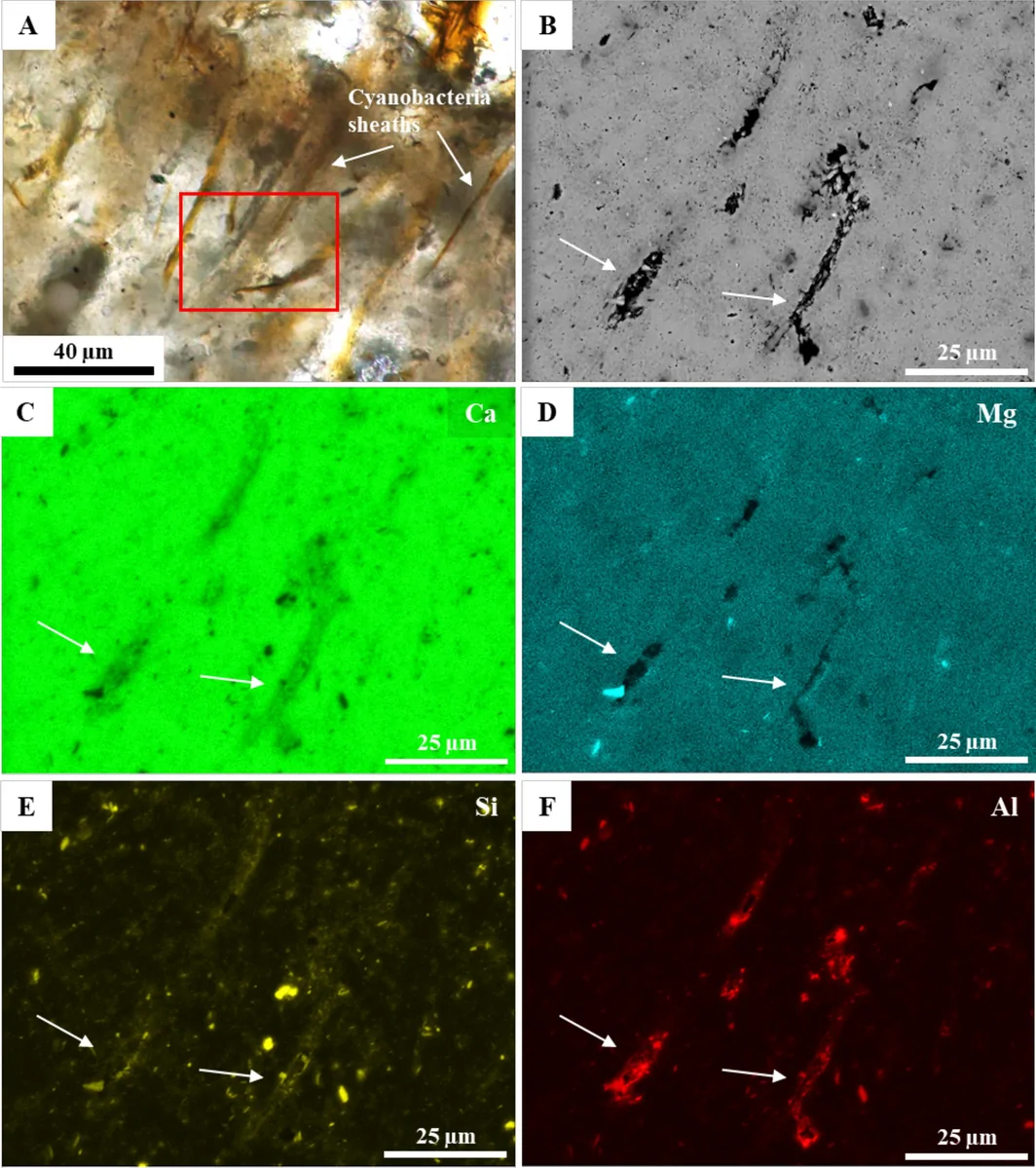
Elemental characterisation of filamentous microstructures in Carri Laufquen stromatolites. (A) Optical photomicrograph showing filamentous microstructures filled with yellow‐brown material (white arrows). (B–E) SEM image (B) and EDS maps showing the distributions of (C) Ca, (D) Mg, (E) Si and (F) Al in the matrix and in association with filamentous microstructures. Scale bar in B also applies to C–F.

**FIGURE 7:**
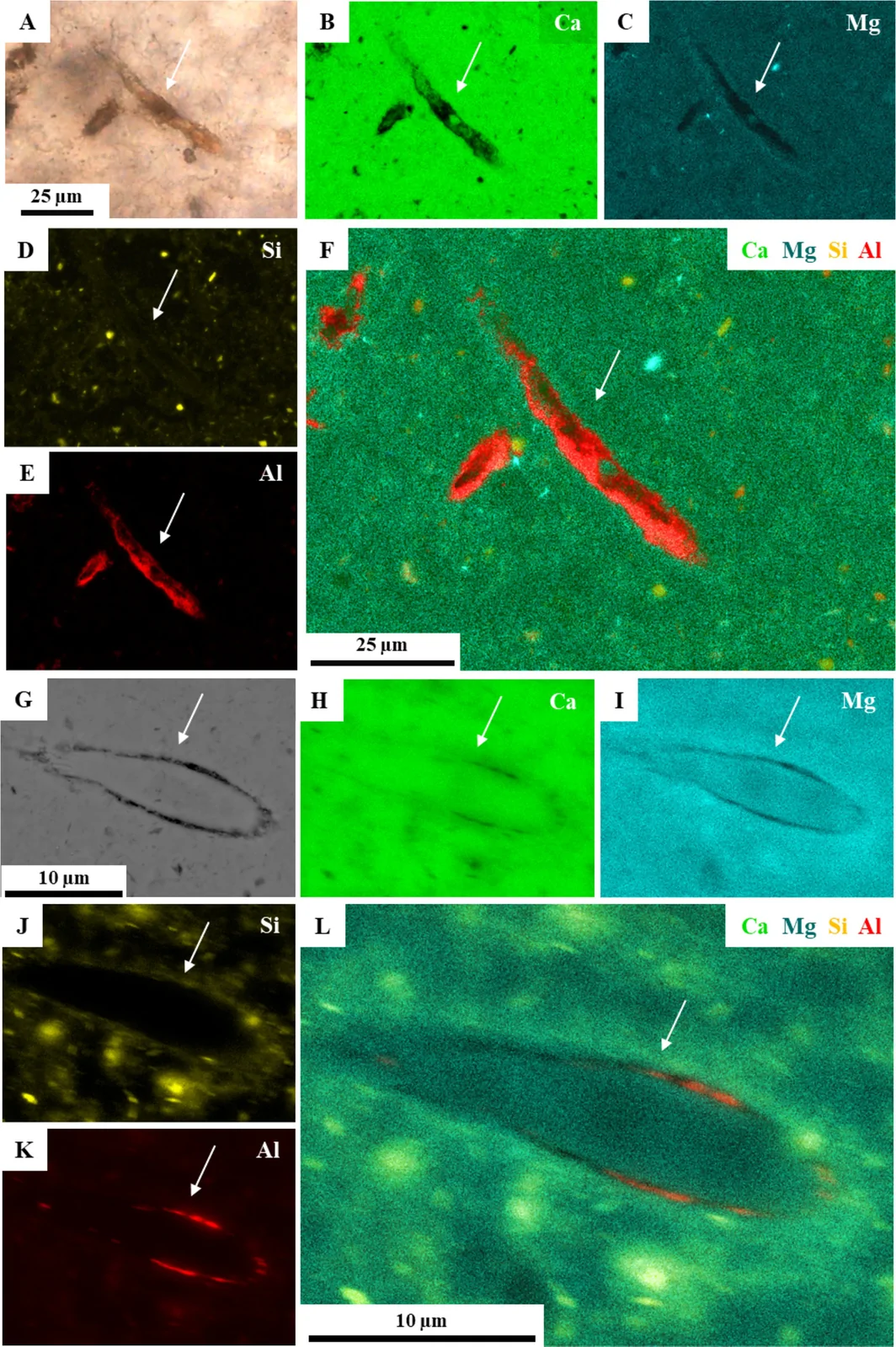
Elemental characterisation of filamentous microstructures in Carri Laufquen stromatolites. (A) Optical photomicrograph showing a longitudinal view through a filamentous microstructure (arrowed). (B–E) EDS elemental maps showing the distributions of (B) Ca, (C) Mg, (D) Si and (E) Al. Scale bar in A applies to B–E. (F) Composite EDS map of filamentous structures showing associations of Ca, Mg, Si and Al. (G) EDS images of an area of interest through a transversal section of a filamentous microstructure (arrowed). (H–K) EDS elemental maps showing the distributions of (H) Ca, (I) Mg, (J) Si and (K) Al. Scale bar in G applies to H–K. (L) Composite EDS map showing associations of Ca, Mg, Si and Al.

SEM‐EDS elemental mapping confirmed that the stromatolite matrix is composed predominantly of carbonate, whereas the filamentous microstructures are enriched in Si and Al, indicating the presence of an Al‐bearing silicate phase (Figure 6). Longitudinal cross‐sections through filamentous microstructures reveal interiors with Al‐rich silicate compositions (Figure 7A–F), whereas transverse cross‐sections reveal Al concentrations in the filament walls (Figure 7G–L).

**FIGURE 8:**
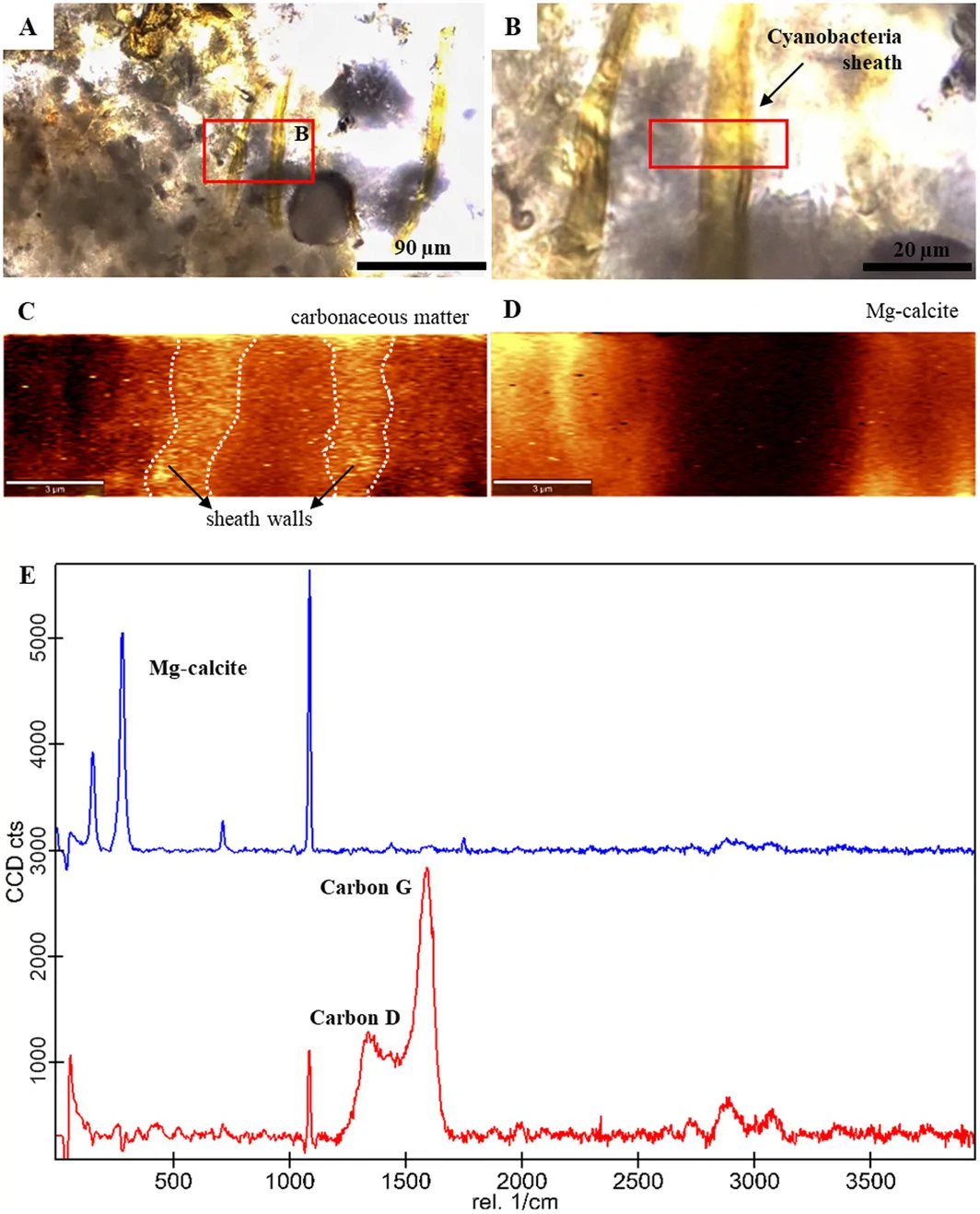
Raman microspectroscopic characterisation of Carri Laufquen filamentous fmicrostructures. (A, B) Optical photomicrographs showing filamentous microstructures. The red square in B represents the area of interest of the microspectroscopic maps. (C) Raman compositional map showing the distribution of Mg‐calcite. (D) Raman compositional map showing the distribution of carbonaceous matter. (E) Representative Raman spectra of Mg‐calcite (blue) and carbonaceous matter (red).

Raman microspectroscopy indicates that the filamentous microstructures contain carbonaceous material (CM) within their walls, identified by characteristic D (1342 cm^−1^) and G (1590 cm^−1^) bands, together with weak second‐order signals in the ~2800–3100 cm^−1^ region (Figure 8). Raman spectral analysis (Figure 8E) further confirms the composition of the low‐Mg calcite matrix, indicated by peaks at 157, 284, 714, and 1088 cm^−1^ (*cf*. Frezzotti et al. 2012).

### 3.3 Microbial Biosignatures

**FIGURE 9:**
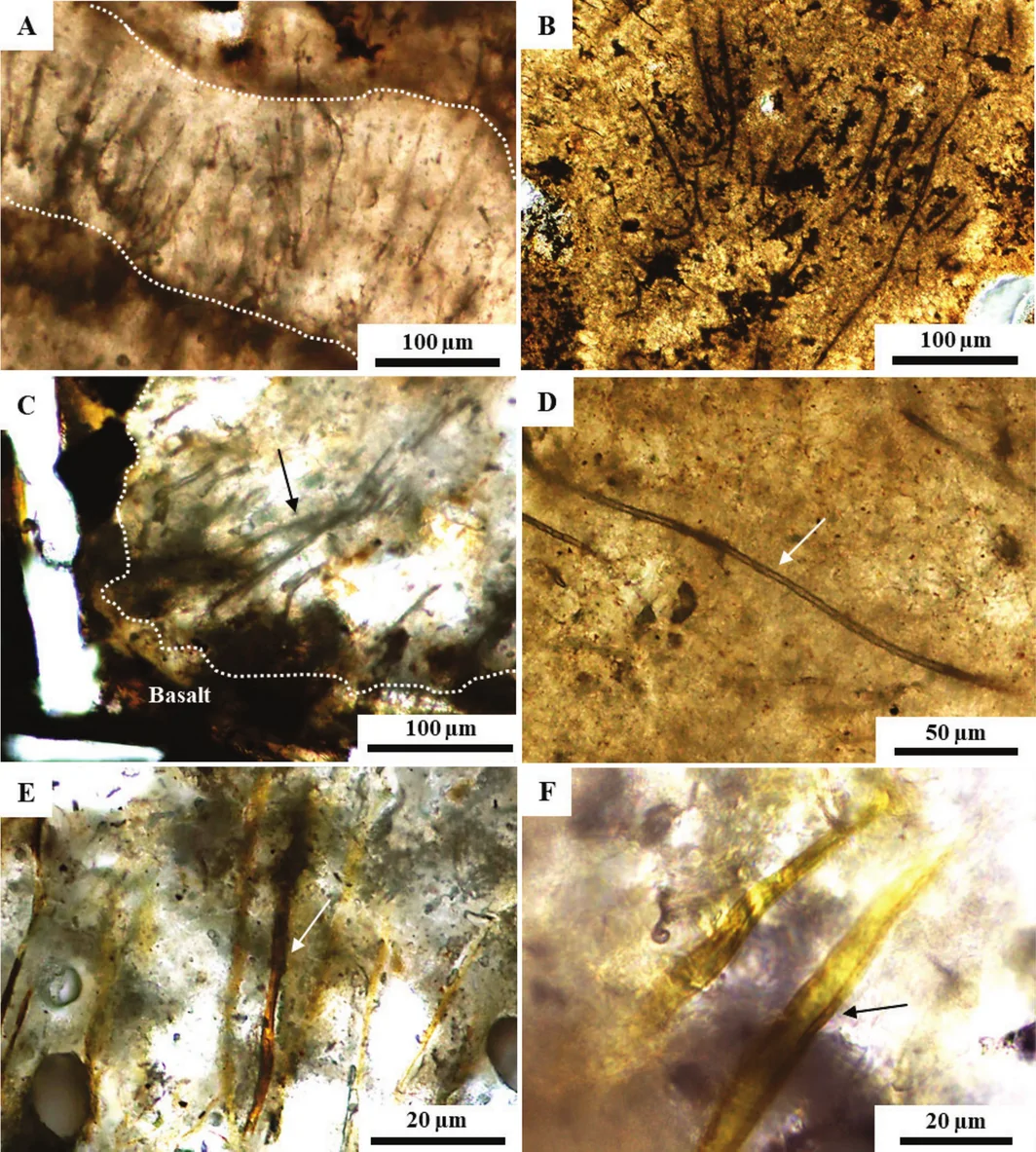
Optical photomicrographs showing distinct forms of filamentous cyanobacterial microstructures preserved within the Carri Laufquen stromatolites. (A) Filamentous cyanobacterial sheaths oriented perpendicular to stromatolitic layering in a (sub‐)parallel arrangement. Filamentous microstructures are associated with microsparite layers (bounded by dashed white lines) in the laminated microsparite microfacies. (B) Cluster of cyanobacterial sheaths within the micritic columnar microfacies. (C) Cluster of cyanobacterial sheaths (arrowed) growing perpendicular to the basaltic substrate (dashed white line denotes the basalt–carbonate interface). (D) Cyanobacterial sheath occurring as an isolated empty mould (arrowed). (E) Filamentous structures filled with a yellow‐brown phase (arrowed). (F) High‐magnification image showing filamentous structures with exceptional preservation of sheath walls (arrowed).

Optical microscopy of Carri Laufquen stromatolites reveals moderately to well‐preserved filamentous microstructures, which are interpreted as fossilised cyanobacterial sheaths (*cf*. Eymard et al. 2021; Figure 9). These straight to gently curved, non‐septate, unbranched filamentous microstructures occur predominantly within microsparitic laminae and exhibit two diameter populations: narrower structures measuring 3–5 μm and wider structures up to 8.5 μm in diameter. The narrower structures cannot be unambiguously interpreted from optical microscopy alone and may represent collapsed sheaths, whereas the wider structures are consistent with preserved sheaths. Filamentous microstructures are either isolated or clustered, locally forming palisade‐like fabrics or, more rarely, exhibiting random orientations (Figure 9A,B). Some occur in direct contact with the basalt substrate, oriented perpendicular to the carbonate–basalt interface (Figure 9C). The wider sheaths are preserved either as empty moulds or infilled by a yellow‐brown phase (Figure 9D,E); the latter likely corresponding to the Al‐bearing silicate phase identified by SEM‐EDS (Figures 6 and 7). In many cases, the sheath walls are exceptionally well preserved (Figure 9F).

**FIGURE 10:**
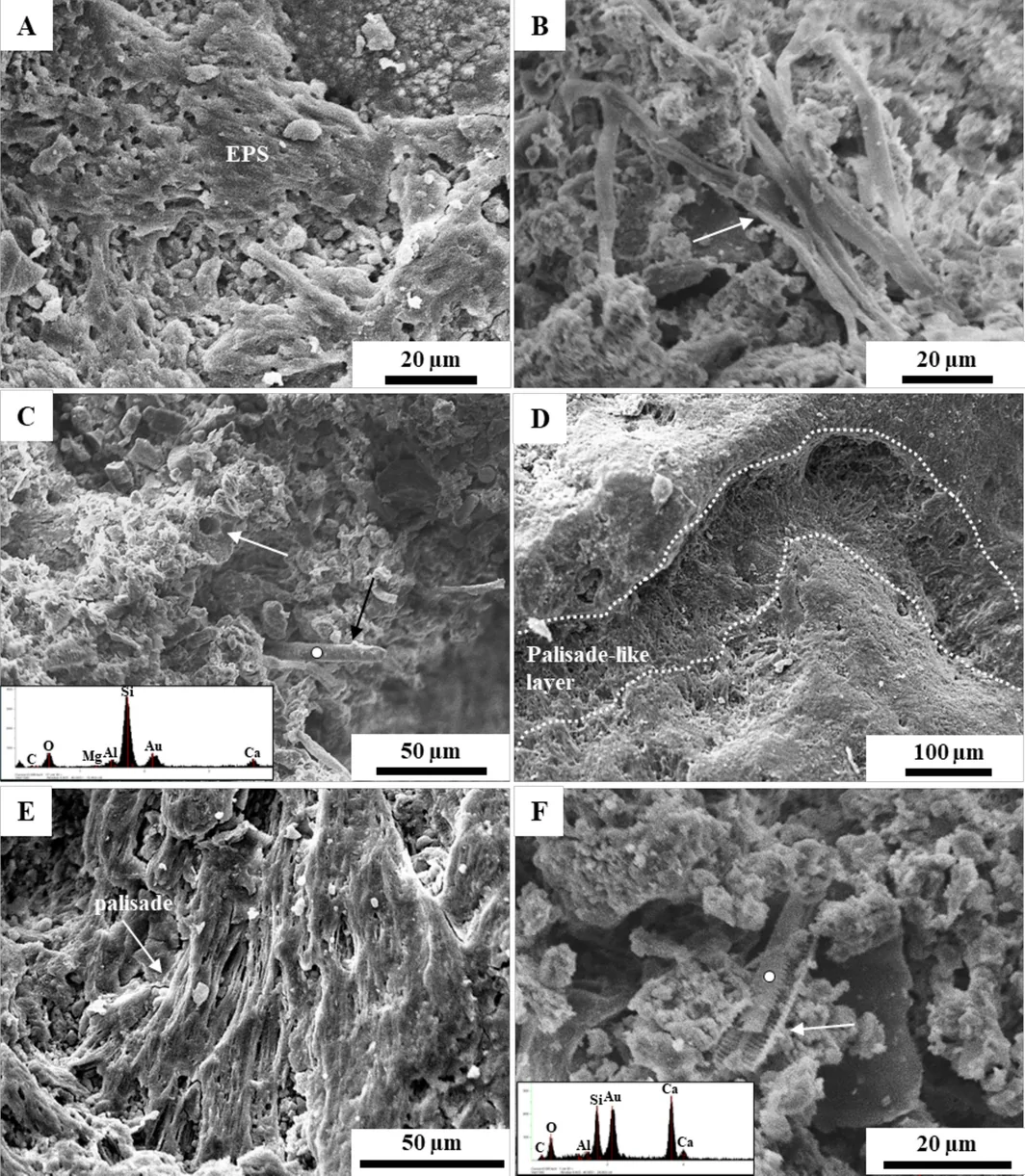
SEM micrographs showing the microbial components of the Carri Laufquen stromatolites. (A) Amorphous biofilm in the matrix, interpreted as extracellular polymeric substances. (B) Cluster of filamentous cyanobacterial sheaths (arrowed) with diameters of approximately 3–5 μm. (C) Empty mould (white arrow) interpreted as the relic of a possible filamentous structure (example indicated with black arrow). The composition of this filamentous structure is rich in Si, O and Al; see inset EDS spectrum. (D) Palisade‐like texture represented by a cluster of filamentous cyanobacterial structures, which are oriented vertically and perpendicular to layering between two micritic laminae. (E) Close‐up of filamentous palisade‐like texture. (F) Fragmented diatom frustule trapped in the matrix. Note that Au in EDS spectra comes from the 10 nm Au coating applied prior to analysis.

SEM imaging of Carri Laufquen stromatolites also reveals fossilised cyanobacterial sheaths in spatial association with other microbial components, including extracellular polymeric substances (EPS), preserved as biofilm surrounding the sheaths (Figure 10A,B). Sheath moulds are occasionally preserved as empty cavities or infilled by a poorly crystalline Al‐bearing silicate phase (Figure 10C). Sheaths commonly form densely packed, vertically aligned clusters, producing palisade‐like textures primarily within microsparitic laminae (Figure 10D,E). Fragmented or partially dissolved diatom frustules are also observed in association with the carbonate matrix or embedded within microbial EPS (Figure 10F).

**FIGURE 11:**
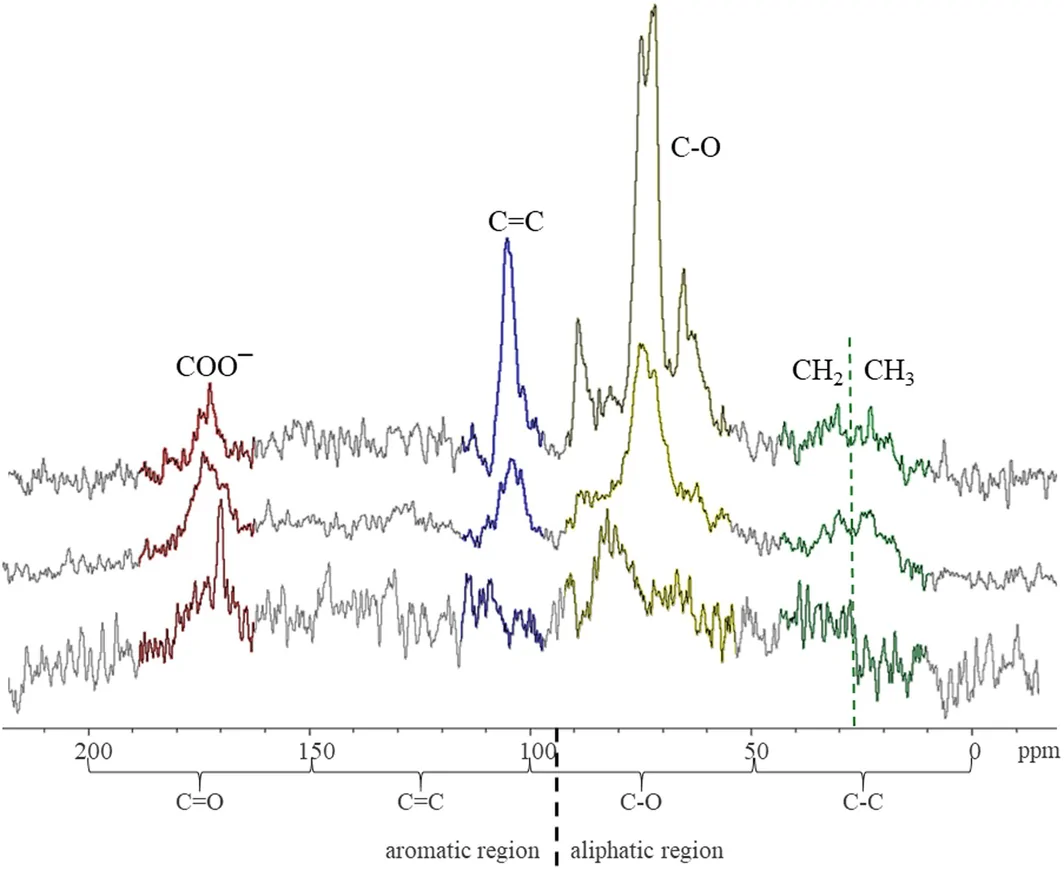
Solid state ^13^C NMR spectra of Carri Laufquen stromatolites. Three representative spectra showing the range of organic groups identified according to their ^13^C NMR intensity, including aliphatic carbon (CH_2_, CH_3_), carbon–oxygen (C–O), unsaturated carbon (C=C) and carboxylic acid (COO^−^) moieties.

Solid state ^13^C NMR of powdered stromatolite samples confirms the presence of biomolecular components in the Carri Laufquen stromatolites (Figure 11). Spectra show broad signals resonating between δ 10 and 185 ppm, dominated by peaks from aliphatic carbon (CH_3_ resonating at ~24 ppm, CH_2_ at ~31 ppm), C–O groups resonating at ~75 ppm, unsaturated C=C resonating at ~110 ppm, and carboxylic acids resonating at ~175 ppm. These results confirm and extend the complexity in carbonaceous material compositions identified using Raman spectroscopy (Figure 8).

**FIGURE 12:**
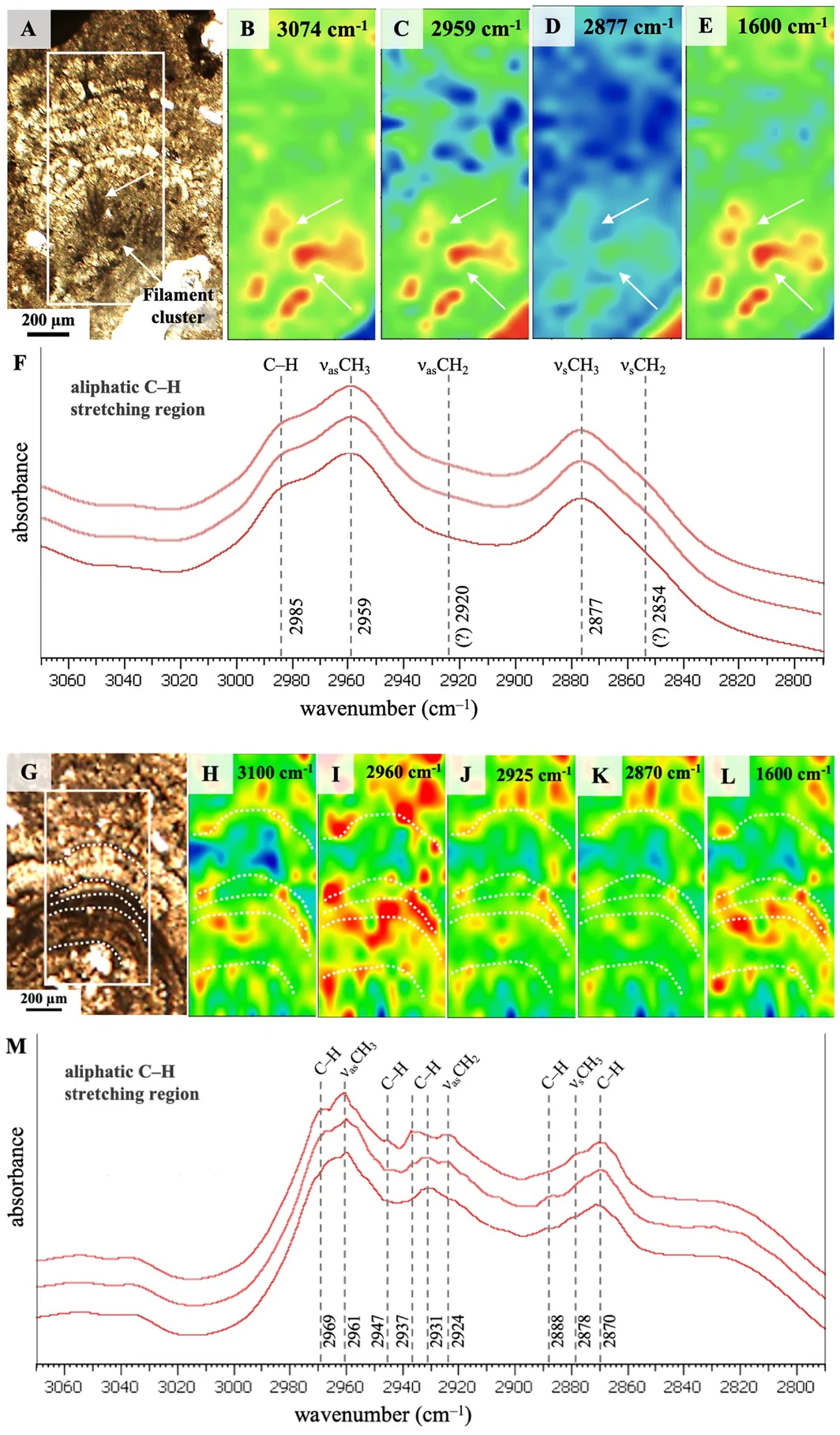
FTIR microspectroscopy characterisation of organic‐rich samples. (A) Optical photomicrograph showing a region dominated by filamentous structures. (B–E) FTIR microspectroscopic maps showing aliphatic C–H and aromatic C=C moieties, which are concentrated in the filament‐rich region. (F) Representative FTIR spectra showing the aliphatic stretching region. (G) Optical photomicrograph showing microbial laminations (white dotted lines). (H–L) FTIR microspectroscopic maps showing aliphatic C–H and aromatic C=C moieties (see text for details). (M) Representative FTIR spectra showing the aliphatic stretching region.

FTIR microspectroscopic mapping of stromatolite fabrics shows strong organic signals in both filamentous microstructures (Figure 12A–F) and microbial laminations (Figure 12G–M), spatially resolving the presence of aromatic C=C, C–H and aliphatic compounds identified using ^13^C NMR spectroscopy. The 3000–2800 cm^−1^ region reveals distinct CH_3_ stretches (~2870 and ~2960 cm^−1^) in microbial laminae and CH_3_ and CH_2_ stretches (~2920–2925 cm^−1^) in filamentous structures, confirming the preservation of aliphatic organic moieties.

## 4 Discussion

### 4.1 Growth of Carri Laufquen Stromatolites

Stromatolites from the Maquinchao Basin were collected at CLG, CLC and along the shores of the Maquinchao River (Figure 2). Their distinct macromorphologies (Figure 3) were not restricted to any specific lake or outcrop, indicating that stromatolite form is not controlled by locality alone.

The stromatolites grew directly onto and around basaltic substrates, including pebbles, cobbles and boulders, with individual stromatolitic crusts reaching up to 50 cm in diameter (Figures 2 and 3). These occurrences are similar to other lacustrine stromatolites decribed from the East African Rift System (e.g., Moussa et al. 2023; Dorneles et al. 2024, 2026), where interactions between basalt weathering, hydrothermal inputs and alkaline waters promoted the geochemical conditions required for the formation of millimetre‐ to centimetre‐scale stromatolitic carbonates. Although stromatolite morphologies arise from complex organism–sediment–environment interactions (e.g., Pace et al. 2018), their macroscopic architecture is primarily governed by environmental conditions (Suosaari et al. 2016). Stromatolite accretion fundamentally depends on microbial biofilms that trap sediments and induce carbonate precipitation (Riding 2011), with the resulting morphology reflecting the interplay between microbial growth and environmental factors.

Eymard et al. (2019)—also studying the Maquinchao Basin—noted that basaltic clasts of similar size may support different stromatolitic morphologies, suggesting that factors other than substrate size, such as local water depth or hydrodynamic conditions, also influence stromatolite development. Our observations are consistent with this interpretation, as no relationship was observed between stromatolitic macromorphologies and the size of the basaltic nucleus onto which the carbonate precipitation initiated. Stromatolite formation is further controlled by the stability of the water level under intrinsic and extrinsic conditions (Dupraz et al. 2006; Eymard et al. 2019). Stable, low‐energy and low‐turbidity lacustrine environments promote the development of large‐scale stromatolites (e.g., Figure 3A–D), whereas thinner stromatolitic crusts (e.g., Figure 3I,J) likely reflect water level instability, possibly indicating brief highstands followed by regression.

At the microscale, the Carri Laufquen stromatolites are dominated by laminated microcolumnar fabrics composed of alternating micritic and microsparitic laminae (Figure 4). These microcolumnar fabrics are independent of macromorphology and represent the fundamental building blocks of the carbonate framework.

Laminated microfabrics comprise both laterally continuous, isopachous radial‐fibrous low‐Mg calcite laminae and irregular, non‐isopachous micritic laminae (Figure 4A,B). The former likely reflect a stronger influence of physicochemical carbonate precipitation (*cf*. Grotzinger and Knoll 1999), whereas the irregular micritic laminae, containing abundant filamentous microbial structures and biofilm remnants (Figure 9), indicate that microbial growth also contributed to stromatolite accretion. In modern microbialites, lamination commonly develops through sediment trapping and binding by microbial mats together with microbially mediated carbonate precipitation (Frantz et al. 2015; Suosaari et al. 2016). In contrast, many Precambrian stromatolites are interpreted to reflect alternating micritic laminae associated with organic matter and sparry carbonate formed predominantly through physicochemical precipitation (Riding 2011). The laminated microfabrics observed in the Carri Laufquen stromatolites more closely resemble the latter model, in which microbial communities provide the template for stromatolite accretion while physicochemical precipitation contributes substantially to lamination development (Barbieri 2026). This highlights the value of the Carri Laufquen Lakes System for investigating processes relevant to ancient stromatolite formation.

Pacton et al. (2015) reported that the mineralogical composition of low‐Mg‐calcite in the Carri Laufquen stromatolites remains consistent across different layers; however, in situ analyses using TIMA‐EDS and μXRD revealed a distinction between low‐Mg calcite and high‐Mg calcite, which correlates with colour variation in successive stromatolitic laminae. Specifically, dark laminae contain predominantly high‐Mg calcite whereas bright laminae comprise mostly low‐Mg calcite (Figure 5). Although the darker appearance of the micritic laminae in thin section is influenced by their finer crystal size, these laminae also preserve higher concentrations of organic compounds than the adjacent microsparitic laminae, as demonstrated by FTIR microspectroscopy (Figure 12G–M). This distribution is consistent with the model of Dupraz et al. (2004), in which early micritic precipitation occurs within EPS‐rich microbial biofilms, whereas later microsparitic cement precipitating through physicochemical processes preserves comparatively little organic matter.

EPS macromolecules rich in hydroxyl and carboxyl groups provide highly reactive nucleation sites that strongly bind with Ca^2+^ and Mg^2+^ cations, leading to the formation of carbonate crystals (Dupraz and Visscher 2005). In Carri Laufquen samples, coupling results from FTIR and TIMA‐EDS analyses suggest a particularly close association between elevated magnesium concentrations in Mg‐carbonate minerals and the presence of microbial organic material. This relationship supports that microbial EPS can preferentially bind Mg^2+^ and provide nucleation sites for carbonate precipitation (*cf*. Dupraz et al. 2004). Experimental work by Zhang et al. (2023) further demonstrated that biomediated carbonates become enriched in MgCO_3_ because EPS‐rich biofilms promote Mg^2+^ adsorption or incorporation into the calcite lattice while inducing particle aggregation.

### 4.2 Evidence for Microbial Influence on Carbonate Precipitation

Optical and electron microscopy observations provide compelling evidence for the preservation of microbial communities within the Carri Laufquen stromatolites, including abundant isolated and clustered cyanobacterial sheaths (Figure 9), EPS, biofilms, and diatom frustules (Figure 10).

The frequent occurrence of densely packed and vertically oriented clusters of filamentous microfossils forming palisade‐like textures (Figures 9A and 10D,E) indicates that many microbial components were preserved in situ and in life position, demonstrating their close association with stromatolite growth (Castro‐Contreras et al. 2014; Hickman‐Lewis et al. 2019). The observed palisade texture is consistent with the expected growth patterns of photosynthetic microorganisms, particularly cyanobacteria, which orient themselves toward sunlight to maximise and sustain their metabolic activity (e.g., Álvaro et al. 2021). Similar fabrics have been reported from both modern and fossil lacustrine stromatolites, including actively growing stromatolites from the Maquinchao River (Pacton et al. 2015; Eymard et al. 2020), supporting the interpretation that cyanobacterial communities were closely associated with stromatolite formation and that carbonate organomineralisation in the Maquinchao Basin reflects microbial influence on lamination development. Although photosynthetic activity may have influenced local carbonate precipitation through modifications of microenvironmental chemistry (Dupraz et al. 2004, 2009; Pace et al. 2018), this mechanism cannot be demonstrated directly from the present observations.

In carbonate microbialites, organomineralisation begins with the precipitation of nanospheroidal crystals of low‐Mg calcite in association with EPS, which serve as organic matrices for the nucleation and formation of micritic laminae (Figure 10D), followed by the growth of larger crystals. This process continues until the final stage of organomineralisation, marked by the nucleation of microsparite laminae. Such laminae are commonly associated with palisade‐like textures in our samples (Figure 10E). Mineralisation continues until there is no remaining EPS to be degraded. This mechanism of lamina formation, a function of the availability of Ca^2+^ and Mg^2+^ ions, also controls the development of thicker or thinner micritic laminae within microcolumns (*cf*. Petryshyn et al. 2012), as observed in the TIMA phase maps (Figure 5).

SEM observations of the Carri Laufquen stromatolites revealed widespread mineralised EPS (Figure 10A) and the preservation of filamentous cyanobacterial sheaths (Figure 10B), indicating that microbial mediation was a dominant mechanism driving mineral formation. Similar processes have been reported in Middle Miocene stromatolites from the North Alpine Foreland Basin (Mörsingen, Germany), where sustained CaCO_3_ precipitation led to the shape‐retentive mineralisation of cyanobacterial sheaths and the preservation of palisade‐like textures (Willmer and Rasser 2022).

### 4.3 Biosignature Preservation within Carri Laufquen Stromatolites

The Carri Laufquen stromatolites preserve multiple lines of evidence for biological activity, including well‐preserved cyanobacterial sheaths and biofilm EPS, and moulds of filamentous sheaths. These moulds occur either as empty structures (Figures 9D and 10C) or infilled with a poorly crystalline Al‐bearing silicate phase (Figures 6, 7, 10C). Similar associations of amorphous Mg‐silicates, carbonates and EPS were documented in the Maquinchao Basin by Pacton et al. (2015), who proposed microbial mediation as the fundamental driver of their precipitation. However, they did not identify Al‐rich phases and attributed the minimal amount of Si detected to diatom dissolution. In contrast, our analysis revealed both fragmented diatom frustules as a potential Si source (Figure 10F) and the common presence of Al (identified using EDS) forming localised amorphous Al‐silicate phases that infill cyanobacterial filamentous moulds (Figures 6, 7 and 10C).

The recognition of localised Al‐bearing silicate phases was facilitated by the combined use of high spatial resolution SEM‐EDS elemental mapping and Raman microspectroscopy, which enabled the characterisation of mineral–organic associations at the scale of individual microbial structures. Unlike bulk mineralogical analyses or lower‐resolution approaches, these techniques resolve microscale spatial relationships between phases, providing new evidence for complex mineralisation pathways involved in the preservation of microbial biosignatures in the Carri Laufquen stromatolites.

Raman microspectroscopy (Figure 8) reveals concentrations of organic matter within cyanobacterial sheath walls that spatially coincide with Si–Al‐enriched zones identified using EDS mapping (Figure 7G–L), indicating a close association between Al‐rich silicates and carbonaceous matter. Poorly crystallised Al‐Mg‐bearing silicate deposits in alkaline environments have previously been linked to microbial activity (Arp et al. 2003, in Satonda Crater Lake, Indonesia; Souza‐Egipsy et al. 2005, in Mono Lake, USA; Dorneles et al. 2026, in Lake Ashenge, Ethiopia; and Mercedes‐Martín et al. 2025, in Lake Clifton, Australia). Fiore et al. (2011) suggested that Al‐rich silicate phases may form from the precipitation of an aluminosilicate gel—promoted by microbial organic products, including EPS—with subsequent crystallisation driven by metabolically induced changes in the chemistry of the surrounding microenvironment. In these cases, elevated concentrations of Si‐Al in sheath walls (Figure 7) likely promoted their exceptional preservation, as observed in the Carri Laufquen stromatolites (Figures 8 and 9).

The precursor to these Al‐bearing silicate phases remains uncertain. Based on experimental studies (Bontognali et al. 2014), they may initially precipitate as poorly crystalline aluminosilicate gels associated with EPS before undergoing progressive crystallisation. In comparable alkaline lacustrine environments, such early precipitates have been linked to the formation of Mg‐Al‐bearing clay minerals (e.g., Pace et al. 2016; Cupertino et al. 2024; Dorneles et al. 2026), and Mg‐bearing silicate phases have also been reported from the Maquinchao Basin (Pacton et al. 2015). Nevertheless, the available SEM‐EDS and Raman data do not permit a definitive mineralogical assignment, and additional mineralogical analyses would be required to determine the specific authigenic phase.

Eymard et al. (2020) described Al‐Si phases in samples of modern stromatolites from the Maquinchao Basin, suggesting that this material enhances the preservation of cyanobacterial sheaths and proposing a detrital association for the Al‐bearing silicate phases. Newman et al. (2016) demonstrated experimentally that clay particles play a crucial role in preserving cyanobacterial filaments by rapidly trapping and coating them, conserving their morphology within days under oxic conditions. Similarly, Wacey et al. (2018) reported that silicate‐containing filaments likely represent an early permineralisation stage arising primarily from diagenetic processes. Although the authigenesis of clay mineral phases in lacustrine systems remains incompletely understood, SEM micrographs—coupled with Raman and EDS mapping—provide evidence that authigenic silicate phases in the Carri Laufquen stromatolites may contribute to the preservation of microbial structures and morphologies, as suggested for other systems by Wacey et al. (2018) and Mercedes‐Martín et al. (2025).

Organic geochemical analyses further support the exceptional preservation of fossil biomass in the Carri Laufquen stromatolites. Raman (Figure 8), ^13^C NMR (Figure 11) and FTIR (Figure 12) spectroscopy techniques reveal aromatic and aliphatic carbon moieties and carboxylic functional groups, consistent with the preservation of kerogen (e.g., Marshall et al. 2007; Delarue et al. 2016). Microscopic and geochemical evidence suggests that this organic material originates from the microbial architects of these stromatolites, with cyanobacteria representing a major precursor due to their lipid‐rich cell membranes and high kerogen‐forming potential (Summons et al. 2022). ^13^C NMR spectra from Carri Laufquen (Figure 11) closely resemble those of the cyanobacterium *Synechococcus* (Mackay and Norton 1987) and cyanobacteria‐dominated algal assemblages (Liu et al. 2009), further supporting a cyanobacterial origin for the preserved organic matter and filamentous microstructures. Cyanobacterial cell walls also contain algaenan, an aliphatic‐rich biomacromolecule that is resistant to diagenetic degradation and may play a key role in kerogen formation (e.g., Biller et al. 2015).

FTIR microspectroscopic measurements revealed the preservation of aliphatic C–H moieties, indicating a remarkable degree of organic preservation in the Carri Laufquen stromatolites (Figure 12). The presence of distinct symmetric and asymmetric vibrational stretches from CH_2_ and CH_3_ moieties suggests limited diagenetic influences on primary biochemistry.

Understanding organic–mineral interactions is crucial for reconstructing the poorly understood diagenetic evolution of organic matter (Craddock et al. 2020). Coupled Raman and FTIR analyses allow the in situ characterisation of organic matter within its mineralogical context (Hickman‐Lewis et al. 2024). Nanogeoscience studies also highlight the key role of authigenic K–Al‐phyllosilicates in preserving organic materials by inhibiting organic maturation and protecting diverse biogeochemical signatures, including bacterial and archaeal lipid residues, aromatic compounds, and EPS (Hickman‐Lewis et al. 2025), likely via a similar mechanism to the Al‐silicate phenomena observed in Carri Laufquen stromatolites. Additionally, although ^13^C NMR represents a valuable tool for organic geochemistry, as demonstrated by meteoritics studies (e.g., Cody et al. 2002), very few studies have applied such techniques to carbonate stromatolites. The data presented here demonstrate the potential of ^13^C NMR for investigating biosignature preservation in alkaline lacustrine environments, with implications for both geobiological and astrobiological exploration.

### 4.4 Astrobiological Implications of the Carri Laufquen Stromatolites

Stromatolites preserve long‐term records of microbial activity and organo‐sedimentary interactions, offering critical insights into microbial evolution (Hickman‐Lewis et al. 2019; Hohl and Viehmann 2021). Their capacity to archive both morphological and geochemical information also makes them valuable terrestrial analogues for planetary studies, particularly in the context of exploring ancient Martian palaeoenvironments and detecting putative extraterrestrial biosignatures.

Potential habitable environments on early Mars, particularly during the Noachian–Hesperian, were likely restricted to localised hydrothermal, lacustrine, and subsurface rock‐hosted settings (Hays et al. 2017; Hurowitz et al. 2023). Terrestrial analogues such as the Carri Laufquen Lakes System provide valuable models for understanding how microbial life might have persisted in basalt‐hosted, alkaline lacustrine environments, and the processes by which biosignatures may be preserved over geological timescales (Hays et al. 2017). The present study demonstrates several phenomena that sustain the Carri Laufquen Lakes System as both a palaeoenvironmental and process analogue for Martian palaeolakes, of which Jezero crater is a key example: (1) the presence of Mg‐bearing calcite phases deposited under high carbonate alkalinity conditions; (2) the spatial distribution of Mg‐bearing carbonates, which in both the Carri Laufquen lakes and Jezero crater occur specifically along the shoreline, suggesting deposition at least partly due to shoreline processes; (3) the formation of Mg‐bearing calcite in an alkaline lake hosted within basaltic bedrock; and (4) the gradual drying out of the lake, resulting in falling water levels, the exposure of the carbonate‐bearing shoreline, and the subsequent weathering and erosion of carbonate over geological timescales, which has occurred in both systems, albeit over different timescales. Further to this last point, our results demonstrate the resilience of stromatolite‐forming organisms under fluctuating and increasingly extreme hydrological and diagenetic conditions, a scenario that has parallels on ancient Mars as the surface of the planet neared the end of its habitable period.

At Jezero crater, the NASA Mars 2020 *Perseverance* rover has identified and sampled carbonate‐bearing deposits along the crater margin (Horgan et al. 2020; Tarnas et al. 2021; Williford et al. 2025). The mineral paragenesis of the Carri Laufquen stromatolites, including Mg‐carbonates, minor Al‐silicates, and carbonaceous matter preservation, shares broad similarities with assemblages identified at Jezero crater and other Martian palaeolacustrine sites (e.g., Michalski et al. 2022; Burnie et al. 2023). Furthermore, the preservation of organic material within phyllosilicate matrices, as highlighted in recent studies of ancient terrestrial microbial mats (Hickman‐Lewis et al. 2025), reinforces the potential for long‐term organic preservation in Martian lacustrine settings.

The multi‐technique analytical approach used herein also directly informs the development of strategies for biosignature detection in Martian geological materials and provides a reference framework for laboratory analyses of samples collected on Mars, which will be studied on Earth following Mars Sample Return (MSR). Distinguishing between biogenic and abiotic carbonate structures remains a central challenge to life detection on Mars (Chan et al. 2019; Bosak et al. 2021), and our findings also support that carbonate‐rich lacustrine settings associated with Al‐silicates represent high‐priority targets for addressing such questions while searching for potential biosignatures.

By demonstrating the exceptional fossilisation potential of microbial communities in an environmentally extreme alkaline terrestrial analogue, this study provides critical insights for understanding the preservation pathways of organic material in basalt‐hosted lacustrine environments. Such environments were perhaps the most important and widespread habitable environments on the Noachian–Hesperian Mars (Hurowitz et al. 2023); understanding their terrestrial analogues thus provides critical guidance for astrobiological exploration strategies in Martian sedimentary sequences.

## 5 Conclusions

The Carri Laufquen Lakes System provides a valuable modern analogue for understanding the formation, evolution and preservation of microbial life in alkaline, basalt‐hosted lacustrine environments of relevance to the early Earth and Mars. Stromatolites from this system exhibit distinct morphologies, including shrub‐like, globular, laminated and crust‐like coatings that grew directly over basaltic substrates. The variable morphologies of these stromatolites result from complex interactions between organisms, sediments, and the environment, with lake‐level fluctuations influencing carbonate precipitation. Carbonate microfacies within these stromatolites exhibit diverse laminated and columnar structures indicative of microbial growth. These results demonstrate that cyanobacteria were the principal architects of stromatolite formation at Carri Laufquen, driving carbonate precipitation through metabolic activity and EPS production, and promoting the preservation of filamentous microstructures and organic matter.

Optical and electron microscopy observations reveal abundant microbial remains, including filamentous sheaths and EPS‐rich biofilms, together with phases that document the early stages of organomineralisation and its contribution to microbial fabric preservation. These observations provide clear evidence that early mineralisation processes play a critical role in stabilising microbial fabrics and enhancing biosignature preservation, including both microbial structures and biomolecular components, in lacustrine environments. In particular, the recognition of a close association between microbial structures, organic matter and Al‐rich silicate phases indicates that permineralisation, likely mediated by EPS and microbial metabolism, significantly enhances the retention of both morphological and molecular biosignatures in lacustrine stromatolites. The coupled precipitation of carbonate and authigenic silicate phases under strongly alkaline conditions highlights their significance for high‐fidelity preservation of morphological, molecular and geochemical traces of life.

By integrating microstructural observations with mineralogical and organic geochemical analyses, this study provides a comprehensive framework for understanding the processes governing the preservation of biosignatures in terrestrial lacustrine stromatolites. The Carri Laufquen stromatolites demonstrate that basalt‐hosted alkaline lakes can support resilient microbial ecosystems and promote exceptional fossilisation potential through coupled carbonate and authigenic silicate mineralisation pathways. These findings improve our understanding of the mechanisms responsible for the preservation of microbial biosignatures in both modern and ancient lacustrine systems. Furthermore, because similar basalt‐hosted palaeolacustrine environments existed on early Mars, these results strengthen the interpretation of such settings as high‐priority targets in the search for morphological, molecular and mineralogical biosignatures beyond Earth.

## 6 Materials and Methods

### 6.1 Samples

Samples were collected during a field campaign in March 2023. Outcrops were located based on previous work by Pacton et al. (2015) and Eymard et al. (2019). Sample preparation and analyses took place at the Natural History Museum, UK, and Università di Bologna, Italy. For optical and electron microscopy, Raman and Fourier transform infrared (FTIR) microspectroscopy, and micro X‐ray diffraction (μXRD), 19 samples were embedded in epoxy resin to produce 12 standard uncovered petrographic thin sections (~60 μm thickness and 47 × 27 mm dimensions) and 24 uncovered large‐format thin sections (~60 μm thickness and 60 × 90 mm dimensions). Six of these thin sections were metallised with a 10 nm carbon coating for SEM‐EDS and TESCAN Integrated Mineral Analyzer (TIMA) analyses. Duplicate sections of the same samples were kept uncoated to preserve their suitability for other techniques, including Raman, μXRD, and FTIR.

### 6.2 Optical Microscopy

Microfacies and microbial textures were imaged under transmitted and reflected light using an Olympus BX63 system equipped with a CCD camera (Imaging and Analysis Centre [IAC], NHM London) and a ZEISS Axiophot optical microscope operating at a focal distance of 35 mm, equipped with a Nikon DS‐Fi2 camera (Università di Bologna). Images were acquired using Olympus CellSens and Zeiss AxioVision softwares.

### 6.3 Raman Microspectroscopy

Petrographic observations were complemented by Raman microspectroscopy performed using an Oxford WITec Alpha300 R microscope equipped with a 532 nm green laser (Università di Bologna). Raman spectra were collected using a 100× Nikon objective and a frequency doubled Nd:YAG (532 nm) Ar‐ion 20 mW monochromatic laser source. Beam centring and spectral calibration were performed prior to spectral acquisition using a Si standard (111) with a characteristic Si Raman peak at 520.4 cm^−1^; the optimal power for analysis of different mineral phases was determined experimentally. Raman analyses were visualised and interpreted using WITec Project Management and Image Project Plus software suite and compared with reference spectra from the RRUFF database.

### 6.4 SEM‐EDS

Elemental compositions of selected regions of interest were analysed using scanning electron microscopy equipped with energy dispersive X‐ray spectroscopy (SEM‐EDS) on six representative thin sections and eight freshly fractured pieces of the same six samples. Thin sections were studied using a JEOL JSM‐IT500 SEM equipped with an Oxford Instruments X‐Max EDS detector operating at 15 kV (IAC, NHM London). Freshly fractured pieces of the same samples were gold‐coated for observation using a JEOL JSM‐5400 SEM operating at 15 kV (Università di Bologna).

### 6.5 TIMA

Automated mineralogy analysis was performed using a TESCAN TIMA (IAC, NHM London) instrument equipped with four EDAX Element 30 EDS detectors and operated at a 25 kV accelerating voltage, 13–14 nA probe current and 15 mm working distance. The analysis of a single polished thin section acquired more than 21 million EDS points over a duration of 8 h per sample. Phase map mineralogy was determined by collecting backscattered electron (BSE) maps combined with EDS analysis at a 7 μm pixel resolution with a total of 1000 X‐ray counts per pixel.

### 6.6 μXRD

Micro X‐ray diffraction (μXRD) measurements were performed on five thin sections using a Rigaku D‐max Rapid2 instrument (IAC, NHM London) equipped with a curved 2D imaging plate detector and emitting Cu radiation at 40 kV and 36 mA. The X‐ray beam footprint on the sample measured 300 × 100 μm. Sample loading and centring were carried out using the instrumental microscope. Measurements were performed at a fixed omega angle of 20° 2θ, with data collection durations of 15 min. 2D data were converted into 1D XRD patterns using 2DP software (Rigaku) and all μXRD patterns underwent background subtraction prior to phase identification. Phase identification was performed using HighscorePlus (Panalytical) in conjunction with the PDF‐4 database (International Centre for Diffraction Data; ICDD). The Mg content in calcite was determined using the position of the d104 reflection of calcite, using established crystallographic calibrations (e.g., Mackenzie et al. 1983).

### 6.7 NMR

Solid state ^13^C nuclear magnetic resonance (NMR) spectroscopy was performed to investigate the molecular organic components of ten bulk samples, using a Bruker Avance Neo 400 MHz NMR spectrometer equipped with a 4 mm cross‐polarization magic angle spinning (CP‐MAS) probe at the Slovenian NMR Center (Ljubljana, Slovenia). Samples were powdered (grain size < 10 μm) with the aid of a Dremel micro‐drill instrument (1.6 mm diameter) in order to separate approximately 200 mg from each layer, without mixing materials from distinct microfacies, then treated with HCl (10%) for 48 h to dissolve the inorganic carbonate, thereby concentrating the organic matter for analysis. All samples were spun at the magic angle at 10 kHz and 28°C. ^13^C CP‐MAS NMR experiments resulted in the excitation of protons with a 90° pulse (P1) of 3.5 μs, contact time of 2 ms, and signal acquisition with high‐power proton decoupling. A total of 7168 or 8192 scans were accumulated with a repetition delay of 5 s. Chemical shifts were referenced externally using adamantane.

### 6.8 FTIR

Fourier transform infrared (FTIR) spectral maps of four thin sections were obtained using a Nicolet iN10 mx FTIR microscope (ThermoFisher Scientific) at the IAC, NHM London. Maps covered the areas of interest with a grid spacing of 30 μm in both axes and an aperture size of 50 × 50 μm. Spectra were collected in transmission for 19.73 s (64 scans) over a wavenumber range of 4000–675 cm^−1^ with a spectral resolution of 4 cm^−1^ using a liquid nitrogen‐cooled MCT/A detector and a KBr beamsplitter. A background spectrum with the same parameters was collected through air every 20 min. Single spectra were acquired for 78.92 s (256 scans) with the aperture set to 50 × 50 μm. A background spectrum with the same parameters was collected through air prior to each single spectrum acquisition. Data were acquired and processed using OmnicPicta software (ThermoFisher Scientific).

## Funding

This work was supported by UK Space Agency, ST/V00560X/1, ST/Z000491/1; Space it Up!, 2024‐5‐E.0; Agence Nationale de la Recherche, LIBIOPANE ANR‐23‐CE49‐0001; Europlanet, 21‐EPN‐FT1‐020, 654208; CERIC‐ERIC, 20222015.

## Conflicts of Interest

The authors declare no conflicts of interest.

## Data Availability

The data that support the findings of this study are available from the corresponding author upon reasonable request.

## References

[gbi70063-bib-0001] Aguilera, E. , E. Mazzoni , and J. Rabassa . 2018. “Patagonian Cenozoic Magmatic Activity.” In Volcanic Landscapes and Associated Wetlands of Lowland Patagonia, 31–67. Springer International Publishing.

[gbi70063-bib-0002] Alvarez, M. P. , E. Carol , I. Eymard , A. Bilmes , and D. Ariztegui . 2022. “Hydrochemistry, Isotope Studies and Salt Formation in Saline Lakes of Arid Regions: Extra‐Andean Patagonia, Argentina.” Science of the Total Environment 816: 151529. 34758343 10.1016/j.scitotenv.2021.151529

[gbi70063-bib-0003] Álvaro, J. J. , M. Sánchez‐Román , K. G. Nierop , and F. Peterse . 2021. “Multiscale Microbial Preservation and Biogeochemical Signals in a Modern Hot‐Spring Siliceous Sinter Rich in CO_2_ Emissions, Krýsuvík Geothermal Field, Iceland.” Minerals 11, no. 3: 263.

[gbi70063-bib-0004] Arp, G. , A. Reimer , and J. Reitner . 2003. “Microbialite Formation in Seawater of Increased Alkalinity, Satonda Crater Lake, Indonesia.” Journal of Sedimentary Research 73, no. 1: 105–127.

[gbi70063-bib-0006] Barbieri, R. 2026. “Cyanobacteria as Drivers of Surface Micromorphology.” Bollettino Della Societa Paleontologica Italiana 65, no. 1: 2.

[gbi70063-bib-0007] Biller, P. , A. B. Ross , and S. C. Skill . 2015. “Investigation of the Presence of an Aliphatic Biopolymer in Cyanobacteria: Implications for Kerogen Formation.” Organic Geochemistry 81: 64–69.

[gbi70063-bib-0008] Bontognali, T. R. , F. Martinez‐Ruiz , J. A. McKenzie , A. Bahniuk , S. Anjos , and C. Vasconcelos . 2014. “Smectite Synthesis at Low Temperature and Neutral pH in the Presence of Succinic Acid.” Applied Clay Science 101: 553–557.

[gbi70063-bib-0009] Bosak, T. , A. H. Knoll , and A. P. Petroff . 2013. “The Meaning of Stromatolites.” Annual Review of Earth and Planetary Sciences 41: 21–44.

[gbi70063-bib-0010] Bosak, T. , K. R. Moore , J. Gong , and J. P. Grotzinger . 2021. “Searching for Biosignatures in Sedimentary Rocks From Early Earth and Mars.” Nature Reviews Earth & Environment 2, no. 7: 490–506.

[gbi70063-bib-0011] Bradbury, J. P. , M. Grosjean , S. Stine , and F. Sylvestre . 2001. “Full and Late Glacial Lake Records Along the PEP 1 Transect: Their Role in Developing Interhemispheric Paleoclimate Interactions.” In Interhemispheric Climate Linkages, 265–291. Academic Press.

[gbi70063-bib-0012] Buongiorno, J. , F. J. Gomez , D. A. Fike , and L. C. Kah . 2019. “Mineralized Microbialites as Archives of Environmental Evolution, Laguna Negra, Catamarca Province, Argentina.” Geobiology 17, no. 2: 199–222. 30548907 10.1111/gbi.12327

[gbi70063-bib-0013] Burne, R. V. , and L. S. Moore . 1987. “Microbialites: Organosedimentary Deposits of Benthic Microbial Communities.” PALAIOS 1: 241–254.

[gbi70063-bib-0014] Burnie, T. M. , I. M. Power , C. Paulo , et al. 2023. “Environmental and Mineralogical Controls on Biosignature Preservation in Magnesium Carbonate Systems Analogous to Jezero Crater, Mars.” Astrobiology 23, no. 5: 513–535. 36944136 10.1089/ast.2022.0111

[gbi70063-bib-0015] Çağatay, M. N. , E. Damcı , G. Bayon , and M. Sarı . 2024. “Microbialites on the Northern Shelf of Lake Van, Eastern Türkiye: Morphology, Texture, Stable Isotope Geochemistry and Age.” Sedimentology 71, no. 3: 850–870.

[gbi70063-bib-0016] Cartwright, A. , J. Quade , S. Stine , K. D. Adams , W. Broecker , and H. Cheng . 2011. “Chronostratigraphy and Lake‐Level Changes of Laguna Cari‐Laufquén, Río Negro, Argentina.” Quaternary Research 76, no. 3: 430–440.

[gbi70063-bib-0017] Castro‐Contreras, S. I. , M. K. Gingras , E. Pecoits , et al. 2014. “Textural and Geochemical Features of Freshwater Microbialites From Laguna Bacalar, Quintana Roo, Mexico.” PALAIOS 29, no. 5: 192–209.

[gbi70063-bib-0018] Chan, M. A. , N. W. Hinman , S. L. Potter‐McIntyre , et al. 2019. “Deciphering Biosignatures in Planetary Contexts.” Astrobiology 19, no. 9: 1075–1102. 31335163 10.1089/ast.2018.1903 PMC6708275

[gbi70063-bib-0019] Cody, G. D. , C. O. D. Alexander , and F. Tera . 2002. “Solid‐State (^1^H and ^13^C) Nuclear Magnetic Resonance Spectroscopy of Insoluble Organic Residue in the Murchison Meteorite: A Self‐Consistent Quantitative Analysis.” Geochimica et Cosmochimica Acta 66, no. 10: 1851–1865.

[gbi70063-bib-0020] Craddock, P. R. , A. Haecker , K. D. Bake , and A. E. Pomerantz . 2020. “Universal Curves Describing the Chemical and Physical Evolution of Type II Kerogen During Thermal Maturation.” Energy & Fuels 34, no. 12: 15217–15233.

[gbi70063-bib-0021] Cupertino, D. F. , C. W. Ramnani , M. D. Vanden Berg , and S. M. Awramik . 2024. “Formation of Magnesium‐Clay in a Lacustrine Microbialite‐Bearing Carbonate Deposit, Eocene Green River Formation, Sanpete County, Utah.” Sedimentology 71, no. 1: 263–292.

[gbi70063-bib-0022] Delarue, F. , J. N. Rouzaud , S. Derenne , et al. 2016. “The Raman‐Derived Carbonization Continuum: A Tool to Select the Best Preserved Molecular Structures in Archean Kerogens.” Astrobiology 16, no. 6: 407–417. 27186810 10.1089/ast.2015.1392 PMC4900230

[gbi70063-bib-0023] Dorneles, V. A. C. , K. Hickman‐Lewis , R. Barbieri , A. M. Caminiti , and B. Cavalazzi . 2024. “Microbe‐Mineral Interactions in the Microstromatolitic Crusts of the Lacustrine Chimneys and Volcanic Bedrock of Lake Abhe, Republic of Djibouti.” Bollettino Della Societa Paleontologica Italiana 63, no. 3: 229–244.

[gbi70063-bib-0024] Dorneles, V. A. C. , K. Hickman‐Lewis , T. H. Haileselasie , et al. 2026. “Stromatolites From Lake Ashenge, Ethiopia: Controls on Biosignature Preservation in Extreme Alkaline Environments.” Astrobiology 26, no. 1: 10–29. 41572780 10.1177/15311074251413230

[gbi70063-bib-0025] Dupraz, C. , R. Pattisina , and E. P. Verrecchia . 2006. “Translation of Energy Into Morphology: Simulation of Stromatolite Morphospace Using a Stochastic Model.” Sedimentary Geology 185, no. 3–4: 185–203.

[gbi70063-bib-0026] Dupraz, C. , R. P. Reid , O. Braissant , A. W. Decho , R. S. Norman , and P. T. Visscher . 2009. “Processes of Carbonate Precipitation in Modern Microbial Mats.” Earth‐Science Reviews 96, no. 3: 141–162.

[gbi70063-bib-0027] Dupraz, C. , and P. T. Visscher . 2005. “Microbial Lithification in Marine Stromatolites and Hypersaline Mats.” Trends in Microbiology 13, no. 9: 429–438. 16087339 10.1016/j.tim.2005.07.008

[gbi70063-bib-0028] Dupraz, C. , P. T. Visscher , L. K. Baumgartner , and R. P. Reid . 2004. “Microbe–Mineral Interactions: Early Carbonate Precipitation in a Hypersaline Lake (Eleuthera Island, Bahamas).” Sedimentology 51, no. 4: 745–765.

[gbi70063-bib-0029] Eymard, I. , M. D. P. Alvarez , A. Bilmes , C. Vasconcelos , and D. Ariztegui . 2020. “Tracking Organomineralization Processes From Living Microbial Mats to Fossil Microbialites.” Minerals 10, no. 7: 605.

[gbi70063-bib-0030] Eymard, I. , M. D. P. Álvarez , A. Bilmes , C. Vasconcelos , C. Thomas , and D. Ariztegui . 2021. “Evolving Controls on Mineralization in Patagonian Microbial Mats as Inferred by Water Chemistry, Microscopy and DNA Signatures.” Latin American Journal of Sedimentology and Basin Analysis 28, no. 2: 133–151.

[gbi70063-bib-0031] Eymard, I. , A. Bilmes , M. D. P. Álvarez , et al. 2019. “Growth Morphologies and Plausible Stressors Ruling the Formation of Late Pleistocene Lacustrine Carbonate Buildups in the Maquinchao Basin (Argentina).” Depositional Record 5, no. 3: 498–514.

[gbi70063-bib-0032] Farías, M. E. , N. Rascovan , D. M. Toneatti , et al. 2013. “The Discovery of Stromatolites Developing at 3570 m Above Sea Level in a High‐Altitude Volcanic Lake Socompa, Argentinean Andes.” PLoS One 8, no. 1: e53497. 23308236 10.1371/journal.pone.0053497 PMC3538587

[gbi70063-bib-0033] Fiore, S. , S. Dumontet , F. J. Huertas , and V. Pasquale . 2011. “Bacteria‐Induced Crystallization of Kaolinite.” Applied Clay Science 53, no. 4: 566–571.

[gbi70063-bib-0034] Frantz, C. M. , V. A. Petryshyn , and F. A. Corsetti . 2015. “Grain Trapping by Filamentous Cyanobacterial and Algal Mats: Implications for Stromatolite Microfabrics Through Time.” Geobiology 13, no. 5: 409–423. 26099298 10.1111/gbi.12145

[gbi70063-bib-0035] Frezzotti, M. L. , F. Tecce , and A. Casagli . 2012. “Raman Spectroscopy for Fluid Inclusion Analysis.” Journal of Geochemical Exploration 112: 1–20.

[gbi70063-bib-0036] Galloway, R. W. , V. Markgraf , and J. P. Bradbury . 1988. “Dating Shorelines of Lakes in Patagonia, Argentina.” Journal of South American Earth Sciences 1, no. 2: 195–198.

[gbi70063-bib-0037] Gomez, F. J. , L. C. Kah , J. K. Bartley , and R. A. Astini . 2014. “Microbialites in a High‐Altitude Andean Lake: Multiple Controls on Carbonate Precipitation and Lamina Accretion.” PALAIOS 29, no. 6: 233–249.

[gbi70063-bib-0073] Grotzinger, J. P. , and A. H. Knoll . 1999. “Stromatolites in Precambrian Carbonates: Evolutionary Mileposts or Environmental Dipsticks?” Annual Review of Earth and Planetary Sciences 27, no. 1: 313–358. 10.1146/annurev.earth.27.1.313 11543060

[gbi70063-bib-0038] Hays, L. E. , H. V. Graham , D. J. Des Marais , et al. 2017. “Biosignature Preservation and Detection in Mars Analog Environments.” Astrobiology 17, no. 4: 363–400. 28177270 10.1089/ast.2016.1627 PMC5478115

[gbi70063-bib-0039] Hickman‐Lewis, K. , B. Cavalazzi , K. Giannoukos , et al. 2023. “Advanced Two‐and Three‐Dimensional Insights Into Earth's Oldest Stromatolites (ca. 3.5 Ga): Prospects for the Search for Life on Mars.” Geology 51, no. 1: 33–38.

[gbi70063-bib-0040] Hickman‐Lewis, K. , B. Cavalazzi , and W. Montgomery . 2024. “Correlative Microspectroscopy of Biogenic Fabrics in Proterozoic Silicified Stromatolites.” Geochemical Perspectives Letters 30: 34–39.

[gbi70063-bib-0042] Hickman‐Lewis, K. , J. Cuadros , K. Yi , et al. 2025. “Aluminous Phyllosilicates Promote Exceptional Nanoscale Preservation of Biogeochemical Heterogeneities in Archaean Siliciclastic Microbial Mats.” Nature Communications 16, no. 1: 2726. 10.1038/s41467-025-57727-4 PMC11923192 40108154

[gbi70063-bib-0043] Hickman‐Lewis, K. , P. Gautret , L. Arbaret , et al. 2019. “Mechanistic Morphogenesis of Organo‐Sedimentary Structures Growing Under Geochemically Stressed Conditions: Keystone to Proving the Biogenicity of Some Archaean Stromatolites?” Geosciences 9, no. 8: 359.

[gbi70063-bib-0044] Hohl, S. V. , and S. Viehmann . 2021. “Stromatolites as Geochemical Archives to Reconstruct Microbial Habitats Through Deep Time: Potential and Pitfalls of Novel Radiogenic and Stable Isotope Systems.” Earth‐Science Reviews 218: 103683.

[gbi70063-bib-0045] Horgan, B. H. , R. B. Anderson , G. Dromart , E. S. Amador , and M. S. Rice . 2020. “The Mineral Diversity of Jezero Crater: Evidence for Possible Lacustrine Carbonates on Mars.” Icarus 339: 113526.

[gbi70063-bib-0046] Hurowitz, J. A. , D. C. Catling , and W. W. Fischer . 2023. “High Carbonate Alkalinity Lakes on Mars and Their Potential Role in an Origin of Life Beyond Earth.” Elements 19, no. 1: 37–44.

[gbi70063-bib-0048] Jones, B. , and R. W. Renaut . 2008. “Cyclic Development of Large, Complex, Calcite Dendrite Crystals in the Clinton Travertine, Interior British Columbia, Canada.” Sedimentary Geology 203, no. 1–2: 17–35.

[gbi70063-bib-0049] Kah, L. C. , J. K. Bartley , and A. F. Stagner . 2009. “Reinterpreting a Proterozoic Enigma: Conophyton‐Jacutophyton Stromatolites of the Mesoproterozoic Atar Group, Mauritania.” In Perspectives in Carbonate Geology: A Tribute to the Career of Robert Nathan Ginsburg, 277–295. Blackwell Publishing Ltd.

[gbi70063-bib-0072] Liu, Z. , J. Mao , M. L. Peterson , C. Lee , S. G. Wakeham , and P. G. Hatcher . 2009. “Characterization of Sinking Particles From the Northwest Mediterranean Sea Using Advanced Solid‐State NMR.” Geochimica et Cosmochimica Acta 73, no. 4: 1014–1026.

[gbi70063-bib-0050] Mackay, M. A. , and R. S. Norton . 1987. “13C Nuclear Magnetic Resonance Study of Biosynthesis of Glucosylglycerol by a Cyanobacterium Under Osmotic Stress.” Microbiology 133, no. 6: 1535–1542.

[gbi70063-bib-0051] Mackenzie, F. T. , W. D. Bischoff , F. C. Bishop , M. Loijens , J. Schoonmaker , and R. Wollast . 1983. “Magnesian Calcites; Low‐Temperature Occurrence, Solubility and Solid‐Solution Behavior.” Reviews in Mineralogy and Geochemistry 11, no. 1: 97–144.

[gbi70063-bib-0052] Marshall, C. P. , G. D. Love , C. E. Snape , et al. 2007. “Structural Characterization of Kerogen in 3.4 ga Archaean Cherts From the Pilbara Craton, Western Australia.” Precambrian Research 155, no. 1–2: 1–23.

[gbi70063-bib-0053] Mercedes‐Martín, R. , M. Sánchez‐Román , C. Ayora , et al. 2025. “Formation of Mg‐Silicates in the Microbial Sediments of a Saline, Mildly Alkaline Coastal Lake (Lake Clifton, Australia): Environmental Versus Microbiological Drivers.” Sedimentology 72, no. 5: 1518–1547.

[gbi70063-bib-0054] Michalski, J. R. , T. A. Goudge , S. A. Crowe , J. Cuadros , J. F. Mustard , and S. S. Johnson . 2022. “Geological Diversity and Microbiological Potential of Lakes on Mars.” Nature Astronomy 6, no. 10: 1133–1141.

[gbi70063-bib-0055] Moussa, N. , G. Bayon , V. Dekov , et al. 2023. “Mixed Carbonate‐Siliceous Hydrothermal Chimneys Ahead of the Asal Propagating Rift (SE Afar Rift, Republic of Djibouti).” Journal of African Earth Sciences 197: 104765.

[gbi70063-bib-0056] Newman, S. A. , G. Mariotti , S. Pruss , and T. Bosak . 2016. “Insights Into Cyanobacterial Fossilization in Ediacaran Siliciclastic Environments.” Geology 44, no. 7: 579–582.

[gbi70063-bib-0058] Pace, A. , R. Bourillot , A. Bouton , et al. 2016. “Microbial and Diagenetic Steps Leading to the Mineralisation of Great Salt Lake Microbialites.” Scientific Reports 6, no. 1: 31495. 27527125 10.1038/srep31495 PMC4985759

[gbi70063-bib-0057] Pace, A. , R. Bourillot , A. Bouton , et al. 2018. “Formation of Stromatolite Lamina at the Interface of Oxygenic–Anoxygenic Photosynthesis.” Geobiology 16, no. 4: 378–398. 29573198 10.1111/gbi.12281

[gbi70063-bib-0059] Pacton, M. , G. Hunger , V. Martinuzzi , et al. 2015. “Organomineralization Processes in Freshwater Stromatolites: A Living Example From Eastern P Atagonia.” Depositional Record 1, no. 2: 130–146.

[gbi70063-bib-0060] Petryshyn, V. A. , F. A. Corsetti , W. M. Berelson , W. Beaumont , and S. P. Lund . 2012. “Stromatolite Lamination Frequency, Walker Lake, Nevada: Implications for Stromatolites as Biosignatures.” Geology 40, no. 6: 499–502.

[gbi70063-bib-0061] Riding, R. 2011. “The Nature of Stromatolites: 3,500 Million Years of History and a Century of Research.” In Advances in Stromatolite Geobiology, 29–74. Springer Berlin Heidelberg.

[gbi70063-bib-0062] Souza‐Egipsy, V. , J. Wierzchos , C. Ascaso , and K. H. Nealson . 2005. “Mg–Silica Precipitation in Fossilization Mechanisms of Sand Tufa Endolithic Microbial Community, Mono Lake (California).” Chemical Geology 217, no. 1–2: 77–87.

[gbi70063-bib-0074] Summons, R. E. , P. V. Welander , and D. A. Gold . 2022. “Lipid Biomarkers: Molecular Tools for Illuminating the History of Microbial Life.” Nature Reviews Microbiology 20, no. 3: 174–185. 34635851 10.1038/s41579-021-00636-2

[gbi70063-bib-0063] Suosaari, E. P. , R. P. Reid , P. E. Playford , et al. 2016. “New Multi‐Scale Perspectives on the Stromatolites of Shark Bay, Western Australia.” Scientific Reports 6, no. 1: 20557. 26838605 10.1038/srep20557 PMC4738353

[gbi70063-bib-0064] Tarnas, J. D. , K. M. Stack , M. Parente , et al. 2021. “Characteristics, Origins, and Biosignature Preservation Potential of Carbonate‐Bearing Rocks Within and Outside of Jezero Crater.” Journal of Geophysical Research: Planets 126, no. 11: e2021JE006898. 10.1029/2021JE006898 PMC8597593 34824965

[gbi70063-bib-0065] Tatur, A. , R. del Valle , M. M. Bianchi , et al. 2002. “Late Pleistocene Palaeolakes in the Andean and Extra‐Andean Patagonia at Mid‐Latitudes of South America.” Quaternary International 89, no. 1: 135–150.

[gbi70063-bib-0066] Wacey, D. , L. Urosevic , M. Saunders , and A. D. George . 2018. “Mineralisation of Filamentous Cyanobacteria in Lake Thetis Stromatolites, Western Australia.” Geobiology 16, no. 2: 203–215. 29318763 10.1111/gbi.12272

[gbi70063-bib-0067] Walter, M. R. , R. Buick , and J. S. R. Dunlop . 1980. “Stromatolites 3,400–3,500 Myr Old From the North Pole Area, Western Australia.” Nature 284, no. 5755: 443–445.

[gbi70063-bib-0068] Whatley, R. C. , and G. C. Cusminsky . 1999. “Lacustrine Ostracoda and Late Quaternary Palaeoenvironments From the Lake Cari‐Laufquen Region, Rio Negro Province, Argentina.” Palaeogeography, Palaeoclimatology, Palaeoecology 151, no. 1–3: 229–239.

[gbi70063-bib-0069] Williford, K. H. , K. A. Farley , B. H. Horgan , et al. 2025. “Carbonated Ultramafic Igneous Rocks in Jezero Crater, Mars.” Science 391: 6787. 10.1126/science.adu8264 41405541

[gbi70063-bib-0070] Willmer, B. J. , and M. W. Rasser . 2022. “Calcification Patterns of Rivularia‐Type Cyanobacteria: Examples From the Miocene of the North Alpine Foreland Basin.” Facies 68, no. 4: 16.

[gbi70063-bib-0071] Zhang, C. , F. Li , J. Lyu , and Y. Yao . 2023. “Biomimetic Mineralization of Ca–Mg Carbonates: Relevance to Microbial Cells and Extracellular Polymeric Substances.” Microscopy and Microanalysis 29, no. 2: 665–674. 37749716 10.1093/micmic/ozac044

